# The application of inferior vena cava filters in orthopaedics and current research advances

**DOI:** 10.3389/fbioe.2022.1045220

**Published:** 2022-11-21

**Authors:** Jingchao He, Zhitao Wang, Yue Xin Zhou, Hongbo Ni, XiaoHanu Sun, Jian Xue, Shanshan Chen, Shuai Wang, Meng Niu

**Affiliations:** ^1^ China Medical University, Shenyang, China; ^2^ The First Affiliated Hospital of China Medical University, Shenyang, Liaoning, China; ^3^ Institute of Metal Research, Chinese Academy of Sciences (CAS), Shenyang, Liaoning, China

**Keywords:** deep vein thrombosis, pulmonary embolism, orthopaedic, biodegradable inferior vena cava filter, biodegradable metal material

## Abstract

Deep vein thrombosis is a common clinical peripheral vascular disease that occurs frequently in orthopaedic patients and may lead to pulmonary embolism (PE) if the thrombus is dislodged. pulmonary embolism can be prevented by placing an inferior vena cava filter (IVCF) to intercept the dislodged thrombus. Thus, IVCFs play an important role in orthopaedics. However, the occurrence of complications after inferior vena cava filter placement, particularly recurrent thromboembolism, makes it necessary to carefully assess the risk-benefit of filter placement. There is no accepted statement as to whether IVCF should be placed in orthopaedic patients. Based on the problems currently displayed in the use of IVCFs, an ideal IVCF is proposed that does not affect the vessel wall and haemodynamics and intercepts thrombi well. The biodegradable filters that currently exist come close to the description of an ideal filter that can reduce the occurrence of various complications. Currently available biodegradable IVCFs consist of various organic polymeric materials. Biodegradable metals have shown good performance in making biodegradable IVCFs. However, among the available experimental studies on degradable filters, there are no experimental studies on filters made of degradable metals. This article reviews the use of IVCFs in orthopaedics, the current status of filters and the progress of research into biodegradable vena cava filters and suggests possible future developments based on the published literature by an electronic search of PubMed and Medline databases for articles related to IVCFs searchable by October 2022 and a manual search for citations to relevant studies.

## 1 Introduction

DVT is the abnormal clotting of blood in the deep veins and is a venous return disorder. It is commonly seen in patients who are bedridden and have limited limb movement and is one of the most common peripheral vascular diseases in clinical practice.

Venous thromboembolism (VTE) includes DVT and PE. Acute PE is the most serious clinical manifestation of venous thromboembolism (Konstantinides et al., 2014). When a DVT is dislodged in the body, the embolus travels down the IVC to the right atrium and into the right ventricle, where it reaches the pulmonary artery through the blood flow, causing a serious complication: PE.

The occurrence of PE is substantially associated with untreated lower leg deep vein thrombosis. Currently it is possible to prevent PE by placing an IVCF in the inferior vena cava (IVC) to intercept the dislodged thrombus.

Although all published guidelines agree that IVCFs are indicated in patients who have an acute VTE and who cannot receive anticoagulation medications or in whom adequate anticoagulation has clearly failed despite evidence of appropriate use and effect, some indications are more controversial (Weinberg et al., 2013). There has been considerable debate about whether IVCF is recommended for implantation as a preventive measure for patients in orthopedics (Cohen-Levy et al., 2019).

Currently for orthopaedics, patients undergoing orthopaedic surgery are at increased risk of DVT and PE due to factors such as limited mobility, prolonged bed rest and the use of intraoperative tourniquets, making them a high-risk group for the development of VTE (El-Daly et al., 2013).

The management of thromboprophylaxis in patients with pelvic and acetabular fractures remains a highly controversial topic within the trauma community. Despite anticoagulation, VTE remains the most common cause of surgical morbidity and mortality in this high-risk patient group (El-Daly et al., 2013).

Following surgically treated spinal injuries, the risk of epidural haematoma is low but the consequences are extremely harmful. Although some studies have concluded that well-controlled anticoagulation is not associated with an increased risk of postoperative spinal epidural haematoma (Awad et al., 2005). However, there is still insufficient evidence on the safety of postoperative chemoprophylaxis for patients who have undergone spinal surgery. Therefore, some physicians may be reluctant to start anticoagulation therapy soon after surgery to prevent thrombosis. So some doctors may be reluctant to start anticoagulation shortly after surgery to prevent thrombosis. The impact of anticoagulation therapy on wound healing also makes orthopaedic surgeons cautious about the use of anticoagulants postoperatively (Ploumis et al., 1976). Some surgeons therefore opt for prophylactic implantation of an IVCF to prevent PE and to avoid the risks associated with anticoagulation (Rosner et al., 2004).

However, some studies have now shown that the placement of IVCFs increases the probability of DVT and the long-term benefits of inferior vena cava filters have been questioned (Hirano et al., 2021), raising questions about whether orthopaedic patients would benefit from preoperative IVCF placement.

To perform a more visual assessment of the currently available studies on the inferior vena cava filter, we have visualized and analysed the relevant documentation through vosviewer.

Supplementary Figure S1 shows that the research available on vena cava filters is divided into four main topics: the causes, prevention, and treatment of PE; methods and placement sites of IVCFs in clinical use; complications associated with IVCF implantation; and causes of thrombosis. In Supplementary Figure S2, we can see that in recent years there has been an increased interest in complications related to filter implantation and surgical management.

## 2 Current orthopaedic preventive measures for VTE

In the 2020 Interventional Radiology Society clinical practice guideline for the treatment of patients with venous thromboembolic disease with IVCFs, it was mentioned that given the high efficacy of modern venous thromboembolic drug prophylaxis in surgical patients, future studies should focus on whether certain patients considered to be at high risk of venous thromboembolism (e.g., patients undergoing bariatric, orthopaedic, or cancer surgery) would benefit from IVCF placement (Kaufman et al., 2020).

Patients undergoing orthopaedic procedures, such as total knee arthroplasty (TKA) or total hip arthroplasty (THA), are considered to be at very high risk for the development of venous embolism because of the many factors that contribute to venous stasis, such as the age of the patient, the position on the operating table, the use of a thigh tourniquet to provide a blood-free area during knee arthroplasty, and prolonged resting of the patient before and after surgery.

Before 1980, after a patient underwent orthopaedic surgery, the incidence of symptomatic VTE events was 15–30% in the absence of venous thrombosis prevention, and with modern surgical techniques, postoperative care and effective pharmacological prophylaxis, the rate of symptomatic DVT was estimated at 0.8% and PE at 0.35% (Falck-Ytter et al., 2012).

In one study, it was also noted that the rarity of clinical VTE observation by surgeons may be due to the subclinical nature of coagulation masked by the postoperative inflammatory healing process and the short hospital stay of only a few days in most centres. With shorter hospital stays after major joint surgery, an increasing proportion of patients will be discharged with VTE (Bjørnarå et al., 2006).

A study of medical costs for orthopaedic patients showed that patients with DVT had more than $2,000 more in medical costs compared to patients who did not have DVT (Nutescu et al., 2008).

The above statistics show that patients are at significantly higher risk of developing VTE after all types of orthopaedic surgery, that VTE accounts for a greater proportion of serious adverse outcomes in the postoperative period and that the cost of care for patients with DVT complications after orthopaedic surgery is significantly higher. Therefore, the prevention of pulmonary embolism is particularly important for orthopaedic patients, from both a life and an economic point of view.

There are currently two main types of prevention methods for VTE: post-operative walking, mechanical prophylaxis and chemical prophylaxis.

In addition to reducing the incidence of postoperative thromboembolism, early postoperative ambulation has a positive impact on the patient’s recovery of gastrointestinal function (Talec et al., 2016).

The main drugs commonly used for pharmacological prophylaxis include heparin, factor Xa inhibitors, vitamin K antagonists, direct coagulation inhibitors, and antiplatelets.

Heparin achieves anticoagulation mainly by inhibiting the function of prothrombin (X, IX, XI and XII) after binding to antithrombin (Olson and Chuang, 2002). The common heparin analogues are unfractionated heparin (UFH)and low molecular weight heparin (LMWH). Compared with heparin, LMWH has a longer half-life, and produces a more predictable anticoagulant response (Weitz, 1997).

Factor Xa inhibitors inhibit the production of thrombin by selectively inhibiting factor Xa to achieve anticoagulation (Bauer et al., 2002). Representative drugs include sodium fondaparinux and rivaroxaban. A study in 2019 (Lewis et al., 2019) showed that the use of rivaroxaban was the most effective strategy for preventing deep vein thrombosis in patients undergoing elective TKA.

Vitamin K antagonists inhibit the synthesis of coagulation factors II, VII, IX and X in the liver, which are involved in vitamin K. They are not resistant to coagulation factors II, VII, IX and X that are already present in the blood. Representative drugs include warfarin. In a 2016 guideline (Witt et al., 2016) on warfarin for the treatment of venous thromboembolism, it is stated that warfarin should be started as soon as possible after diagnosis of venous thromboembolism, preferably on the same day, in combination with UFH, LMWH or sulforaphane sodium.

Direct thrombin inhibitors such as dabigatran bind to the fibrin-specific binding site of thrombin and prevent the cleavage of fibrinogen to fibrin, thus blocking the final step in the coagulation waterfall network and thrombosis. This drug is already approved for the prevention of VTE after TKA and THA (Schulman and Majeed, 2011).

Antiplatelets such as aspirin has an antiplatelet effect by inhibiting the production of thromboxane.

The use of these anticoagulants can sometimes be accompanied by some negative effects. For example, the development of heparin-induced thrombocytopenia may occur with the use of heparin (Franchini, 2005), long-term use of aspirin increases the risk of gastrointestinal bleeding (Sostres and Lanas, 2011) and gastric ulcers, and the use of various anticoagulants increases the risk of bleeding (Lewis et al., 2019).

Mechanical prevention mainly includes compression stockings (Hui et al., 1996), external mechanical equipment (Zhao et al., 2014), *etc.* Devices such as compression stockings and external mechanical devices can reduce the chance of DVT by reducing stasis in venous blood flow, but external mechanical devices should not be used in patients with lower limb trauma and compression stockings are contraindicated in patients with peripheral arterial disease and atherosclerosis. These mechanical prophylaxis methods are only external to the body and are non-invasive and do not increase the risk of bleeding compared to pharmacological prophylaxis (Falck-Ytter et al., 2012).

## 3 IVCF for orthopaedic management of VTE

The IVCF is an intravascular interceptor device that intercepts foreign bodies flowing through the IVC and prevents them from reaching the pulmonary artery and causing PE. However, because the function of IVCF is strictly speaking to intercept thrombus and not to prevent thrombosis, IVCF has not been included in many studies as a mechanical prophylactic device. Because of the various risk factors for VTE in orthopaedic patients, IVCF is increasingly being used in the perioperative period in orthopaedic surgery, and the risk benefit of IVCF placement in orthopaedic patients is gaining attention, and the question of whether IVCF should be placed in patients undergoing orthopaedic surgery is beginning to be investigated.

However, these studies have shown mixed results, with some suggesting that vena cava filters may be beneficial in patients undergoing orthopaedic surgery (Stein et al., 2018), and others suggesting that IVCF implantation is unnecessary in orthopaedic patients (Segon et al., 1995). Therefore, determining which patients may not benefit from prophylactic filters and limiting unnecessary placement is one of the challenges of using inferior vena cava filters for prophylactic treatment (Stein et al., 2018).

The incidence of DVT and PE after various types of orthopaedic surgery has been statistically analysed by several researchers. The types of orthopaedic surgery involved in these studies are described separately below as a basis for classification.

### 3.1 The role of IVCF in joint surgery

The incidence of VTE in patients undergoing joint surgery ranges from 2.9% to 3.7% and is the most common postoperative complication after joint surgery (Bjørnarå et al., 2006). The risk of VTE persists for up to 3 months after hip surgery and 1 month after total knee replacement (TKR) (Bjørnarå et al., 2006). For patients who undergo total joint replacement, the incidence of postoperative pulmonary embolism is 1.07%, 81% within the first 3 days, 89% within the first week, and 94% within 2 weeks (Parvizi et al., 2015). For those who underwent joint replacement surgery, fatal pulmonary embolism resulted in 58% of deaths (Dahl et al., 2005).

It is easy to see from the above studies the high incidence and risk of VTE after arthroplasty. Thus, in a 1996 study on the treatment of PE after TJA, the risk of treating symptomatic PE with an IVCF was evaluated and the use of an IVCF for symptomatic PE was shown to be a viable option (Bicalho et al., 1996). This was followed by a 2007 study of arthroplasty patients undergoing IVCF, which again demonstrated that IVCF is a valuable and effective method of preventing fatal thromboembolic outcomes in patients with established thromboembolism (Austin et al., 2007).

However the small number of cases in the two studies mentioned above may have been more biased. Thus, a report in 2021 reviewed 2,857 hip or knee replacements between January 2013 and December 2018. The results showed that for high-risk patients with a history of preoperative VTE the use of an IVCF was associated with a significantly lower incidence of PE and that the incidence of postoperative VTE was more than twice as high in high-risk patients as in other patients. The results demonstrate the effectiveness of prophylactic placement of IVCFs during hip/knee arthroplasty.

In a study comparing approaches to PE prevention after TJA, it was found that among patients who received warfarin, IVCF was associated with fewer complications and lower overall hospital costs compared to the use of heparin for the treatment of PE after TJA (Raphael et al., 2014).

Although there is clear evidence of the efficacy of IVCF in arthroplasty, the complications associated with filter implantation have not been followed over time in the trials described above. In one case report from 2013 (Langlois et al., 2013), it was shown that prolonged placement of the filter led to vena cava thrombosis, which in turn led to intra-articular capsule oedema and ultimately to femoral separation of the prosthesis.

The 9-year cumulative risk of IVC occlusion due to permanent filters is estimated to be 33.2% (Crochet et al., 1999). The fact that IVC occlusion is long term and progressive, even without obvious symptoms, may result in a condition that is not observed by researchers in the short term. Although the use of recyclable filters is now mostly recommended, due to the low retrieval rate of filters, unretrieved filters can remain in the body for long periods of time leading to complications and some patients still choose to have permanent filters placed for various reasons.

Although there is debate as to which is the more effective strategy for the prevention of VTE after TKA, single- or combined-measure prevention strategies (Dorr et al., 2007; Gesell et al., 2013; Lewis et al., 2019). However, the role of IVCF placement in the prevention of PE in a high-risk group with a previous history of VTE after joint surgery is well established.

### 3.2 The role of IVCF in fractures

The studies found on the use of IVCF in fracture patients are mainly in the area of pelvic and acetabular fractures and to a lesser extent in lower limb fractures, the following will focus on pelvic and acetabular fractures and the use of IVCF in these patients.

Patients with pelvic and acetabular fractures have a high probability of developing VTE, with estimates of the incidence of DVT following pelvic trauma ranging from 35% to 61% (Godoy Monzon et al., 2012)and the risk of PE between 2% and 10% (Montgomery et al., 1996). The prevalence of VTE is more in pelvi-acetabular trauma in comparison to hip arthroplasty. This is attributed to multiple factors including high velocity injury, disruption of pelvic vessels, immobilization for long duration and manipulation during surgical procedure (Aggarwal et al., 2020). Comprehensive data show that pulmonary embolism accounts for 10–40% of all deaths in all cases undergoing hip fracture surgery (Dahl et al., 2005).

There are conflicting findings on whether IVCF should be placed in patients with pelvic and acetabular fractures. In two reports in 2005 and 2008, IVCF was placed prophylactically in patients with pelvic and acetabular fractures with preoperative DVT findings and both showed that the filter was safe and effective in preventing PE (Karunakar et al., 2005; Toro et al., 2008).

However, in a recent 2019 study showing a significant increase in prophylactic IVCF insertion during the study period, and the failure of increased prophylactic IVCF use to reduce the incidence of PE or DVT in patients with pelvic and acetabular fractures, it was ultimately concluded that the benefit of prophylactic IVCF placement in this patient population is unclear (Cohen-Levy et al., 2019).

It then becomes a question of whether or not the placement of IVCF will cause pelvic and acetabular fractures in patients. In the guidelines for the prevention of venous thromboembolism in hospitalised patients with pelvic-acetabular trauma, published in 2020, a grading scheme has been devised to classify patients into five categories. Among these, prophylactic IVCF is not recommended for patients in Category 3: Critically ill patient presenting to emergency department (strong recommendation, moderate evidence). For Category 4: Patient with established DVT and planned for surgery placement of IVCF is recommended (weak recommendation, very low evidence) (Aggarwal et al., 2020).

In addition to this, the evidence-based recommendations published in 2021 on the perioperative management of acetabular and pelvic fractures also mention that IVCF cannot be routinely applied prophylactically in patients with pelvic and acetabular fractures, but can be considered in preoperative high-risk groups (Yakkanti et al., 2021).

For patients with lower limb bone fractures, there are fewer relevant studies available.

A 2016 study reported a 2.2% incidence of PE after femur fracture (28/453), with 57.1% of these occurring within 24 h of injury and 89.3% within 48 h of injury (Kim et al., 2016).

In a 1973 study (Fullen et al., 1973), researchers randomly assigned patients diagnosed with traumatic fractures of the proximal femur to either have an IVC filter inserted or no filter inserted. The results showed a lower incidence of PE in patients with a filter placed compared to those without (2% vs. 20%).

In another 2016 report, a retrospective analysis of 2,763 cases of patients with lower limb fractures or pelvic fractures complicated by DVT showed that recyclable IVCF makes a safe and effective approach to prevent PE in patients with fractures complicated by DVT (Pan et al., 2016). In the 2021 report, IVCF was implanted in 964 patients with fractures complicated by DVT. the final results also concluded that the retrievable filter was effective in preventing PE in patients with fractures complicated by DVT (Huang et al., 2021).

From the available studies the placement of IVCF has a significant effect on the prevention of PE in patients with lower limb bone fractures.

### 3.3 The role of IVCF in spine surgery

The incidence of VTE following spine surgery is poorly defined, with reported rates from 0.3% to 31%, suggesting substantial variability in the literature (Kepler et al., 2018). A study in 2018 (Cloney et al., 2018) showed a higher incidence of VTE in patients who underwent spinal fractures compared to those who underwent other spinal procedures.

The difference in postoperative thrombosis prevention strategies for spinal surgery compared to other orthopaedic procedures is the focus on the occurrence of epidural haematomas. Although epidural haematoma is a rare complication the incidence is about 0.2%, epidural haematomas can lead to serious neurological damage (Glotzbecker et al., 1976). Therefore, thromboprophylaxis in spinal surgery requires a balance between the various approaches to thromboprophylaxis and the development of epidural haematomas. Prior to starting treatment, the clinician must consider the appropriate dose, timing and alternatives available to avoid unnecessary complications (Moorthy et al., 2020).

Although some of the current studies have shown that well-controlled anticoagulation is not associated with the risk of spinal epidural haematoma (Awad et al., 2005), some studies have shown that the use of therapeutic doses of heparin is associated with a better risk of bleeding complications (Glotzbecker et al., 1976). However, some studies have also shown that the use of therapeutic doses of heparin results in a better risk of bleeding complications in patients undergoing spinal surgery. There are not enough studies to prove the safety of postoperative chemoprophylaxis for spinal surgery. Some orthopaedic surgeons have therefore chosen to place IVCF.

In a 2005 study (Leon et al., 2005), prophylactic placement of IVCF in 74 spinal surgery patients at high risk of VTE showed that despite the high incidence of postoperative DVT in these spinal surgery patients (23/74), prophylactic placement of IVCF did protect patients from developing PE. However, there was a flaw in this study in that the experiment did not have a control group.

In a 2012 report, a retrospective study of 219 patients who underwent spinal reconstruction surgery with IVCF placement showed that prophylactic placement of IVC filters significantly reduced venous thromboembolism-related events, including PE, compared to group controls. In this study, the incidence of VTE was also found to be significantly higher in patients who received Greenfield permanent filters than in those who received retrievable filters (OR = 2.8, *p* = 0.008).. (McClendon et al., 1976).

In another retrospective study in 2012, in which IVCF was prophylactically placed in 12 patients at high risk of VTE from spinal surgery, a total of 10 patients were eventually retrieved, two of whom had thrombotic entrapment at the time of retrieval. No complications or PEs occurred at subsequent follow-up (Dazley et al., 2012).

In a retrospective study in 2020 on the occurrence of VTE in patients hospitalised after spinal surgery and after discharge, it was shown that IVCF placement was associated with the formation of DVT in hospitalised patients (OR 6.380 [3.414–11.924]) (Cloney et al., 2020). This phenomenon has increased the risk associated with prophylactic placement of IVCF in patients undergoing spinal surgery, making the indications for IVCF placement more limited.

There is no consensus on a postoperative protocol for venous thrombosis prophylaxis after spinal surgery (Alvarado et al., 2020). Recommendations regarding mechanical *versus* chemical prophylaxis vary greatly among institutions. However, in the results of the above-mentioned studies on thromboprophylaxis in patients undergoing spinal surgery, the benefits of prophylactic placement of IVCF in high-risk patients were all shown to be favorable.

### 3.4 Summary of IVCF applications in orthopaedics

Most of the current literature and guidelines suggest that primary prophylaxis with IVCF is not recommended for major surgery in patients without known venous thromboembolism (Falck-Ytter et al., 2012; Mismetti et al., 2015; Kaufman et al., 2020).

This may be due to the problems associated with the prolonged placement of IVCF, which affects the haemodynamics within the IVC and has led to a higher incidence of DVT in patients with filters than in those without filters in some studies (Cloney et al., 2020). This and the occurrence of some other complications of IVCF make the risks of filter placement for the average patient undergoing orthopaedic surgery higher relative to the benefits. So if the filter is to be made more suitable for the general public, there are two improvements.

1. Use retrievable filters as much as possible, retrieve them as soon as possible after the patient is out of the danger period, increase the retrieval rate of filters by active postoperative follow-up and minimise the time they remain in the body.
2. Improve the structure of the filter to reduce the haemodynamic impact of the filter.

In patients at high risk of VTE undergoing orthopaedic surgery, the prophylactic placement of IVCF has been shown in several studies to protect patients and effectively reduce the incidence of PE in patients undergoing all types of orthopaedic surgery.

In summary, the opinion of this article is that IVCF placement is recommended for patients at high risk of VTE who undergo orthopaedic surgery, while for non-VTE orthopaedic patients there is no conclusive data to prove that the benefits of IVCF placement outweigh the risks and it is not recommended.

## 4 IVCF for the prevention of complications of percutaneous vertebroplasty

The function of the IVCF is to intercept foreign bodies in the IVC, so sometimes IVCF will intercept something other than a blood clot, such as bone cement.

Polymethyl methacrylate (PMMA), commonly known as bone cement, is widely used for its biocompatibility, malleability and physical properties to anchor prostheses during joint replacement surgery (Vaishya et al., 2013) and for the treatment of compression fractures, such as percutaneous vertebroplasty (PVP) (Laratta et al., 2017). Although uncommon in procedures such as THA, leakage of PMMA into the circulation is frequently observed during and after vertebroplasty, and PMMA ‘migration’ is the most commonly reported complication of PVP (Yoo et al., 1976).

There are three main mechanisms that cause bone cement to migrate into the venous system: (1) inadequate bone cement polymerization at the time of injection; (2) incorrect needle position; and (3) overfilling of the vertebral body (Baumann et al., 2006). Bone cement leaking outside the vertebral body may reach the IVC along tiny veins, such as the anterior external vertebral plexus, eventually leading to serious conditions such as PE.

### 4.1 Case reports related to filter interception of bone cement

By searching for the role of filters in orthopaedics, we found seven interesting and rare reports of IVCFs intercepting bone cement.

In the first case of IVCF interception of bone cement in 2006 (Herbstreit et al., 1976), a DVT in the femoral vein of the iliac vein was detected during the preoperative examination and a filter was implanted prior to surgery to prevent pulmonary embolism. Post-operatively, it was found that the bone cement from the procedure migrated into the venous blood stream and was captured by the filter, but the surgeon judged that the filter could not be removed conventionally, and the patient subsequently had successful removal of the filter by open surgery.

In the 2009 case (Athreya et al., 2009), during vertebroplasty of L4, the cement leaked into the IVC through the paravertebral vein and it formed a worm-like cast, over which a filter was subsequently placed. Radiographs taken 4 h after the procedure showed that the bone cement had been captured by the filter and that the patient had no corresponding symptoms of cement embolism in the lungs. On the second day after the operation, the surgeon used a Gooseneck snare to successfully capture the embolus and withdraw it into the right common femoral vein, where it was successfully removed by surgery. The patient recovered successfully.

In a 2010 case (Agko et al., 2010), after the discovery of bone cement infiltration into the IVC, no attempt was made to remove the bone cement fragment directly to avoid further damage to the patient, but a Greenfield IVCF was placed over the fragment to prevent embolization. The fragment was subsequently found to be dislodged and captured by the IVCF at follow-up. Initially, the cement fragment was successfully captured by the trap device, but due to the shape of the fragment itself, it could not be retrieved through the sheath. The captured cement fragment was eventually moved to the level of the right common femoral vein and successful retrieval by surgical means.

In the 2015 case report (Edwards et al., 2015), the patient developed DVT during his hospital stay after the first corrective spinal repair. The surgeon placed an IVCF as a means of DVT prophylaxis prior to the second surgery. During the injection of bone cement into the L4 vertebral body during the second procedure, it was discovered that the bone cement had entered the retroperitoneal vein and the injection was stopped. Postoperative observations revealed no complications and the patient recovered function. The filter was then removed 5 months after the operation using an endovascular approach, but the operation was very difficult to perform and took 2 h.

Following the filter retrieval procedure, a CT scan of the patient revealed bone cement in the inferior vena cava where the filter had been placed, presumably during the injection of the bone cement not only had it infiltrated into the paravertebral vein, but some of the bone cement had also reached the IVC and was captured by the filter making the retrieval of the filter unusually difficult.

In a 2013 report (Li et al., 2013), a free thrombus was found in the patient’s left femoral vein on preoperative ultrasound and a filter was implanted prior to PVP to prevent PE. The physician placed a permanent filter due to the patient’s history of malignancy and, referring to the 2010 report (Agko et al., 2010), concluded that the IVCF with intercepted bone cement could not be removed solely by endovascular techniques and that surgical removal would have been extremely risky for this patient and ultimately did not consider removing the IVCF. There were no filter-related complications during the follow-up period of 8 months. In this report, and for the only time to date, the entire process of cement migration into the IVC and capture in the filter was documented intraoperatively by Digital Subtraction Angiography fluoroscopy. This evidence clearly confirms the ability of the filter to trap bone cement and prevent it from migrating into the pulmonary circulation and causing serious damage (Supplementary Figure S3).

In a report from 2021 (Prater et al., 2021), The patient had a retrievable filter placed 2 years ago but not retrieved due to a previous history of DVT. The patient was recently treated with a PVP. She presented several days after her procedure with pain and redness of the skin over the access site. The doctor’s examination revealed that opaque bone cement material was visible along the entire length of the tiny vein extending from the vertebral body to the adjacent inferior vena cava and was intercepted by the filter (Supplementary Figure S4). At the subsequent outpatient follow-up at approximately 2 weeks, the patient reported that the skin changes had subsided, and the patient eventually chose to forgo further intervention in favour of observation.

In the 2022 case report (Han et al., 2022), an IVCF was implanted prior to surgery to prevent PE because of isolated distal DVT in the left calf vein revealed on preoperative ultrasound. Post-operative examination revealed that the bone cement had leaked into the IVC through the paravertebral vein and had become entangled with the filter, making it impossible to remove the filter by conventional means (Supplementary Figure S5). Because of the absence of other symptoms, the patient was eventually discharged from the hospital and given close follow-up and lifelong anticoagulation after discharge for the prevention of secondary IVC and collodion filter thrombosis.

### 4.2 The role of filters in bone cement migration

The anatomical basis for the migration of cement into the IVC lies in the fact that the lumbar veins enter the IVC below the level of the L1-L5 vertebral body and have many connections with the basal vein and segmental veins (Iwanaga et al., 2020) (Supplementary Figure S6). This requires a more accurate understanding of the anatomy of the lumbar veins at the time of surgery in order to avoid bone cement leakage as much as possible. The migration of bone cement observed in the case was through the paravertebral vein into the IVC.

In all of the above cases (Supplementary Table S2), the filter was inserted in the IVC prior to the PVP, except in the 2009 and 2010 cases where the filter was placed specifically to intercept the bone cement after it had migrated into the IVC. What is common to all of these cases is the increased difficulty in retrieving the filter after it has intercepted the bone cement. Some doctors opt out of recycling due to the difficulty of filter recycling. The above cases suggest that in PVP routine pre-operative and post-operative imaging and aggressive intra-operative X-ray fluoroscopy may help in the early diagnosis and treatment of cement leakage.

Because IVCF interception of leaking PMMA is uncommon, there is no recognized optimal remedy, but the aforementioned study demonstrates that IVCF works effectively to intercept leaking bone cement. When a postoperative leak of bone cement into the IVC is identified, if it cannot be removed immediately, consideration may be given to placing a filter over the bone cement and waiting for the debris to dislodge from the IVC wall before finding a way to remove it. In theory, the approach published in 2010 (Agko et al., 2010) is a good alternative if the bone cement does not cling to the filter, but if the bone cement is firmly linked to the filter and the filter itself is permanent, there is no viable solution other than open surgery, as reported in 2006 (Herbstreit et al., 1976).

In 2015 (Guo et al., 2015), it was found that IVCFs had a preventive effect on hypotension and decreased oxygen saturation during the development of cement implantation syndrome in sheep, which were used as experimental subjects to study whether IVCFs could prevent or stop cement implantation syndrome. This finding demonstrates the possible role of IVCFs in high-risk patients undergoing cemented arthroplasty. However, the risk-benefit of IVCF in preventing bone cement implantation syndrome is still unclear and a decision needs to be made on a case-by-case basis at the discretion of the physician.

In 2018 (Isaak et al., 2018), an attempt was made to remove bone cement leaking into the IVC through a “fishing net” technique, which was pretested in a 3D-printed model simulating the patient’s body and was ultimately successful in removing the leaking bone cement from the patient’s IVC. This provides a safe and feasible solution for the removal of bone cement leaking into the IVC (Supplementary Figure S7).

In conclusion, although the interception of leaking bone cement by IVCF is a low probability practice, bone cement leakage is the most common complication of PVP, and it is theoretically unlikely that prophylactic implantation of IVCFs will be used in the future to prevent the serious consequences of bone cement leakage into the IVC because of the difficulty of recycling the filters that intercept the bone cement and the high cost of the filters themselves. However, the successful interception of bone cement by the IVCF in the above case may provide an idea for the prevention of serious consequences of bone cement leaks into the IVC in the future. Is it possible to design a device that is simpler and cheaper, that only needs to have an interception function for a few hours and does not need to be stable in the body for a long time, and that can be recycled together with the leaking bone cement?

## 5 Current status of IVCFs

Initially, IVCFs were mainly made of stainless steel, but due to their mechanical strength, biocompatibility, thermal shape memorability and unsuitability for nuclear magnetic examination, most IVCFs used in clinical practice are now made of nickel-titanium alloy, which is widely used due to its good shape memory effect, superelasticity, corrosion resistance and biocompatibility (Prince et al., 1988). Inferior vena cava filters come in a variety of structures that differ in their capacity to prevent thromboembolism, their effect on haemodynamics, their capacity to maintain stability in the vasculature, and their imaging profile. The inferior vena cava filter is compressed into a delivery sheath at the time of fabrication and can be placed from the femoral, internal jugular, subclavian or anterior elbow veins, releasing the filter upon arrival at the designated location, which is usually the IVC below the lower edge of the renal vein opening (Caplin et al., 2011). The implantation of the filter intercepts the thrombus and effectively reduces the incidence of pulmonary embolism.

### 5.1 Classification of IVCFs

There is a wide range of inferior vena cava filters available, which vary in shape, material and design, and these characteristics have a direct impact on their use in clinical practice. Due to the many characteristics of filters, there are various ways of classifying them, but below, we classify them according to whether they can be recycled after being placed in the clinic and how they can be recycled. These classifications are permanent filters, recyclable filters and temporary filters.

Permanent filters were first employed in clinical practice, and they cannot be removed until the vena cava is surgically incised, and therefore, they remain in the IVC for a long time as a foreign body. A series of linked issues (filter tilt, displacement, fracture, *etc.*) have occurred as a result of its long-term installation in the body. Some patients who require long-term filter implantation but do not have the necessary health conditions for a filter retrieval surgery will still use this type of filter, but this filter is currently less commonly used clinically. Representative products of this type of filter are the Mobin-Uddin umbrella filter (Dupont, 1976), Greenfield filter (Kanter and Moser, 1988), Bird’s Nest filter (Roehm, 1984), Vena Tech LGM filter (Crochet et al., 1993), Vena Tech LP filter (Ahmed et al., 2016), Simon nitinol filter (Poletti et al., 1998) and TrapEase filter (Liu et al., 2005).

The temporary filter is designed for short-term implantation and is released in the body with a rod at the closing end leading to the body surface, at the end of which an anti-dislodgement protection device is attached to prevent dislodgement and placement under the skin. The temporary filter has a connecting rod at the end that extends to the surface of the body to keep it stable in the body. There is therefore no barb fixation on the filter arm. Therefore, there is very little endothelial dilation of the vessel wall by the temporary filter, the filter is safer during recycling, the retrieval rate of the filter is high and no special instrumentation is required for recycling. Representative products of this type of filter are Tempofilter (Bovyn et al., 1997) and Angel Catheter (Tapson et al., 2017).

Retrievable filters usually have a fixed barb in contact with the vessel to maintain the normal position of the filter in the body and a retrieval hook at the retrieval end, which can be removed from the vena cava by a catheter technique, reducing the risk of infection without the need for an external device. It is the most widely used IVCF because it does not need to be removed within a specific period of time and can be retained for a long time if needed. Representative products of this type of filter are the Günther Tulip filter (Hoppe et al., 2006), Denali vena cava filter (Hahn, 2015), OptEase filter (Kalva et al., 2011) Celect filter (De Oliveira Leite et al., 2020), ALN filter (Pellerin et al., 2008) and Crux Vena Cava Filter (Murphy et al., 2009).

### 5.2 Recycling of IVCFs

The removal of retrievable filters requires a new percutaneous puncture and retrieval using a retrieval device. This step is technically demanding, and the filter will be more difficult to remove when it is tilted and in contact with the wall. Endothelial coverage is also one of the factors affecting the retrieval success rate of retrievable filters. Endothelial fixation of the IVCF frame in contact with the vessel wall is usually completed 2 weeks after filter insertion, which maintains the stability of the filter in the body but increases the difficulty of retrieving it and the probability of retrieval complications.

When endothelialization is too high, forcing removal can break the vessel, and it can only be kept in the body as a permanent sort of filter. With longer filter retention times, the likelihood of filter-related complications, such as filter tilt, IVC perforation, filter fracture, filter displacement, inferior vena cava thrombotic obstruction, and recurrent DVT or PE, also increases.

There have been many statistical reports on vena cava filter retrieval rates in recent years (Marquess et al., 2008; Minocha et al., 2010; Iliescu and Haskal, 2012; Kalina et al., 2012; Al-Hakim et al., 2014; Sutphin et al., 2015; Inagaki et al., 2016), but most studies have had small sample sizes and extremely variable filter retrieval rates (8%–95%), which may be due to small sample sizes, differences in physician skill levels, and the adequacy of hospital return systems. Nonetheless, it is clear from these studies that IVCF retrieval rates can be significantly improved through more aggressive post-operative visits and the use of more advanced filter retrieval techniques.

### 5.3 Common complications after filter implantation

There are several complications associated with filter implantation, such as filter tilt, filter displacement, inferior vena cava perforation, filter fracture, recurrent thromboembolism, and incomplete expansion of the filter. Of these, filter tilt seems to have the least impact, but the occurrence of filter tilt greatly increases the likelihood of other complications or is a precomplication of other complications (except for incomplete filter expansion).

#### 5.3.1 Filter tilt

Filter tilt is diagnosed when the angle between the central axis of the filter and the longitudinal axis of the IVC exceeds 15°. According to a review of the literature (Singer and Wang, 2011), the tilt of the filter was greater than 5° in approximately 33% of instances, and severe tilt (>15°–20°) occurred in approximately 3%–9% of cases. When the filter is excessively tilted (>15°), the efficacy of thrombus filtration is lowered, and *in vitro* investigations have shown that slanted filters have a lower filtration capacity for smaller thrombi (Günther et al., 2005). Furthermore, when the tilt of the filter is increased, the thrombus trapped at the filter tip promotes the production of *in situ* thrombi in the vena cava wall (Singer and Wang, 2011). (Supplementary Figure S8).

#### 5.3.2 Filter displacement

Filter displacement is described as the filter being more than 10 mm away from the intended point, which is a dangerous complication. When the filter is placed too close to the renal vein, the risk of renal vein embolism or possibly renal failure increases (Janvier et al., 2010). Filter migration to the heart and lungs, which can lead to serious consequences, has also been reported in the past (Gelbfish and Ascer, 1991; Stösslein and Altmann, 1998). Incorrect size selection at the time of placement is one of the reasons for filter migration. (Supplementary Figure S9).

#### 5.3.3 Inferior vena cava perforation

Vena cava perforation is defined when the filter penetrates the vena cava into structures surrounding the IVC wall, such as the aorta, psoas major, duodenum, and kidney. By more than 3 mm (Supplementary Figure S10). IVC perforation is linked to the filter’s longer retention and tilting, with the angle between the barbs on the strainer arm and the vessel wall becoming sharper as the angle between the hook and the vessel wall grows, which could raise the chance of perforation.

#### 5.3.4 Filter fracture

Filter fracture refers to the loss of structural integrity caused by filter shattering (Supplementary Figure S11).

Filter fracture, according to Xin Li et al. (Li et al., 2020), is caused by the IVC’s rhythmic expansion and contraction during the cardiac cycle, subjecting the IVC filter to repetitive mechanical pressure. This mechanical stress eventually leads to wear and subsequent fracture of the cartridge joints. The fragments move with the circulatory system and may damage vital organs.

#### 5.3.5 Recurrent thromboembolism

Most of the current research on the causes of recurrent thromboembolism suggests that it is due to the adverse haemodynamic effects of the filter on the body after the interception of the thrombus by the IVCF, which slows the blood flow and increases the probability of thrombosis. Computational models suggest that filters may lead to stagnation and turbulence in downstream blood flow after thrombus capture, with a significant increase in the probability of thrombosis (Leask et al., 2004) (Supplementary Figure S12).

#### 5.3.6 Incomplete filter expansion

Incomplete deployment of the filter is usually caused by the structure of the filter itself or by the practitioner’s operational error (Supplementary Figure S13). Early filters, such as Greenfield, were used in a small clinical study in which a large gap was found between poorly distributed filter legs in 17 of the 24 filters placed (Sweeney and Van Aman, 1993). When the filter is not fully deployed, it not only has a reduced ability to intercept the thrombus but also has a reduced radial support, which can result in filter displacement or even IVC perforation (Shimoo and Koide, 2020). This problem has been significantly reduced with improvements in filter construction design and materials, with recent studies showing a low rate of incomplete opening in the range of 0.4–0.5% (Li et al., 2020).

## 6 Ideal IVCF

The various vena cava filters currently available are effective in intercepting blood clots to prevent pulmonary embolism, but complications can still occur after implantation. That is why the search for the ideal filter is ongoing. Depending on the role the filter should play and the needs in use, the ideal IVCF should have the following characteristics:

1. High filtering capacity for blood clots in PE prevention
2. Excellent biocompatibility with minimal haemodynamic effects for prevention of recurrent thromboembolism
3. Nonprocoagulant and nonelectrolytic corrosion capability
4. Strong radial support to prevent tilting and shifting
5. Excellent elasticity and compression qualities, allowing recovery of proper dimensions after release in the IVC
6. High mechanical stability, reducing the possibility of filter breakage
7. Ability to decrease vena cava endothelial damage and the occurrence of vena cava perforation
8. Insensitivity to thrombolytic drugs, such as urokinase heparin
9. Nonmagnetic materials that do not interfere with examinations such as MRI
10. Clear visualization under imaging release localization and postoperative review

## 7 Biodegradable IVCF

In recent years, IVCF has been used on a large scale in clinical practice to prevent PE, but there has been a growing interest in the series of problems associated with complications of filter implantation while preventing PE. Data from an article published by the British Society of Interventional Radiology in 2013 showed that 73% of patients with an IVCF implanted had a retrievable IVCF, with 78% of these patients undergoing an attempt to retrieve the filter and 83% experiencing successful removal, with a significantly lower retrieval rate for filters implanted >9 weeks (Uberoi et al., 2013). As the retention time of the filter in the body increases, the probability of complications during filter retrieval also increases (Grewal et al., 2016). For elderly patients and those with coexisting conditions, the filter retrieval rate is significantly lower (Geisbüsch et al., 2012). It can also be complicated by filter breakage and IVC injuries, such as bleeding and entrapment during filter retrieval (Grewal et al., 2016). Biodegradable IVCFs degrade spontaneously in the body, avoiding complications during retrieval and in long-term filter retention.

Biodegradable IVCFs are IVCFs made from biodegradable materials, also known as bioresorbable IVCFs. They can gradually degrade in the body according to a predetermined pattern after passing through the PE risk period, eliminating the need for secondary surgery for removal, reducing the physical burden of patients, and compensating for the occurrence of a series of complications (tilt, displacement, fracture, perforation, and so on) caused by existing filters that cannot be retrieved.

Biodegradable materials are frequently employed in medical sutures, drug release carriers, tissue engineering, and other sectors, such as orthopaedics, maxillofacial surgery, cardiovascular surgery, and plastic surgery (Spitalny, 2006). Yin et al. (2022) used 3D printing technology to investigate the phased degradation of poly-l-actide (PLLA) vascular scaffolds in mice, as well as the alteration of vascular endothelial cells during scaffold degradation, to elucidate the long-term effects of poly-l-actide vascular scaffolds (PLSs) on vascular repair and to demonstrate the potential of PLSs in promoting endothelial function and positive remodelling. Currently, LP Medical’s NeoVas stent (Han et al., 2018) has received State Drug Administration permission for marketing and is believed to have ushered in a new era of coronary intervention. In the field of biliary stents, several types of biliary biodegradable stents have been developed, and some are already in clinical use (Song et al., 2022).

The above examples indicate the excellent potential of biodegradable materials in the medical field, which is progressively becoming a reality.

However, no therapeutic biodegradable IVCFs are currently available. Because of its potential for great performance, the biodegradable vena cava filter has been a prominent focus of research in China and internationally.

### 7.1 Classification of biodegradable IVCFs

According to a review of the literature on biodegradable IVCFs, there are two types of biodegradable filters explored by researchers: completely biodegradable filters and partially biodegradable filters. Completely degradable filters are made of degradable materials and can be completely degraded after reaching an expected time point; partially degradable filters are usually made of a combination of degradable filters and nondegradable metal stents, and the filter is completely degraded after reaching an expected time point, while the remaining part becomes a stent to support the inferior vena cava; partially degradable filters are also called autodeformable IVCFs or convertible IVCFs.

#### 7.1.1 Partially biodegradable filter

Thors, Gao and Novate Medical Ltd. All use metal and biodegradable materials to create partially biodegradable filters. The common feature of these filters is that when the filter is first implanted, the filter itself remains intact using a filter mesh to intercept the thrombus, but as the biodegradable material degrades in the body, the filter transforms, and the mesh structure disappears into a vascular stent in the inferior vena cava.

Thors and Muck (2011), Gao et al. (2011) have progressed to the animal stage but have had problems with severe endothelial hyperplasia and filter displacement, respectively, and both have yet to clarify the process of degradation of material fragments, their destination, and their effect on the organism.

The Sentry Bioconvertible Inferior Vena Cava Filter from Novate Medical Ltd. Was tested in animal trials in May 2018 (Gaines et al., 2018), and its performance was validated against the OptEase filter. In a 1-year analysis of the prospective multicentre SENTRY clinical trial published in August 2018 (Dake et al., 2018), it was shown that by month 12 post-implantation, morphological conversion was completed in 96.4% (106/110) of the filters, and no symptomatic pulmonary embolism was demonstrated at 12 months. In the November 2019 article (Dake et al., 2018), it was shown that 96.5% (82/85) of filters successfully converted morphologically at 24 months in the second year of follow-up, and there was no evidence of late inferior vena cava obstruction or thrombosis following filter conversion at 24 months of follow-up, nor was there evidence of a correlation between filter morphology conversion and thrombosis. In 2020 (Dake et al., 2020), the Sentry Bioconvertible Inferior Vena Cava Filter was clinically implanted in an 85-year-old female patient who did not experience any adverse events within 3 weeks of implantation, and no subsequent adverse events were reported in this patient, making the Sentry filter the first bioconvertible filter to be approved (Supplementary Figure S14).

After the filter cone has entirely dissolved, the semidegradable filter takes on the appearance of a vascular stent. Although not completely degraded *in vivo*, the remaining stent structure has minimal haemodynamic impact and reduces the incidence of recurrent thromboembolism. It also lowers the risk of recurrent thromboembolism, and the undegraded component can be used as a vascular stent to maintain vascular morphology, avoiding subsequent surgery and lowering the risk of problems associated with filter implantation.

#### 7.1.2 Fully biodegradable filter

Zhang, Yang and Eggers have each produced fully biodegradable filters using various biodegradable materials, which are characterized by the fact that the filters degrade in the body over a predicted period of time, ultimately leaving no residual material in the body.

Zhang’s study (Zhang et al., 2017) has progressed to the animal testing phase, with *in vivo* experiments in beagles. The results showed that all filters were mildly displaced, in one case to the right atrium, and that there was an inflammatory reaction in all inferior vena cava (Supplementary Figure S15).

Yang’s study (Yang et al., 2019), which only progressed as far as *in vitro* simulations, confirmed that the filter degraded progressively more rapidly in the cone than in the stent and that the average *in vitro* thrombus capture efficiency of 90% was comparable to that of a conventional descending vein filter. This finding demonstrates the clinical potential of this filter, but the efficacy of this filter *in vivo* is not yet known, as animal studies have not yet been performed, and further studies are needed to investigate the degradation *in vivo*.

Eggers screened Poly (p-dioxanone) (PPDO) as a candidate material for a degradable descending interface filter in 2012 (Eggers and Reitman, 2012). The thrombus interception ability of the filter was initially validated in pigs in 2015 (Eggers et al., 2015) and further validated by *in vitro* simulations in 2016 (Dria and Eggers, 2016). The results suggest that the absorbable filters are likely to be comparable in efficacy to Greenfield filters. In 2017 (Huang et al., 2017), the safety of the absorbable inferior vena cava filter was again demonstrated in pigs, and in 2019 (Eggers et al., 2019)^,^ the filter was compared with the Cook Celect inferior vena cava filter, which demonstrated a good safety profile, particularly with regard to vena cava perforation, as all control metal filters showed perforation. For the first time in 2020 (Elizondo et al., 2020), biodegradable vena cava filters made of PPDO were placed prospectively in eight patients at high risk of lower limb deep vein thrombosis. In this trial, all filters were successfully implanted and intercepted the thrombus with a success rate of 100%, and no pulmonary embolism or filter-related complications occurred.

The above summary shows that the current research on fully degradable filters is based on the use of organic polymers for production, and the production process is mostly based on the use of weaving methods. However, research progress has varied from *in vitro* validation in a simulated environment to animal testing, with Eggers’ research being the most advanced and rapid to date, having progressed to the stage of prospective implantation of filters in humans.

The most serious problem that has arisen thus far in these experiments is the inadequate mechanical properties of some of the filters to maintain radial support in the body, which has led to the displacement of the filters in several experiments. This issue will have a direct impact on the safety of the filter, causing a major safety hazard, and is the largest obstacle on the current path of research into fully degradable filters.

The reason for this phenomenon may be, on the one hand, that the filter itself does not have sufficient mechanical properties to maintain sufficient radial support in the IVC or, on the other hand, that the actual size of the filter is less than the theoretical value due to compression by the compression sheath, and it does not return to its proper size when it is released in the IVC. Consideration can be given to increasing the radial support force directly by changing the structure of the filter so that the filter is fixed in position in the IVC, by directly increasing the mechanical properties of the filter itself by changing the material from which the filter is made, or by increasing the memory and elasticity of the filter so that the filter can return to its proper size after release.

In addition, the use of biodegradable polymers has the unavoidable problem of significantly reducing their ability to develop under radiation compared to metal filters. To solve this problem, the use of biodegradable metallic materials can be considered, or the composition of the material can be changed to increase the developing power of biodegradable polymers.

### 7.2 Increase the imaging capability

There have been numerous attempts by scholars to address the abovementioned situation where fully degradable materials do not develop well under X-ray through various methods. The development of organic polymer compounds with enhanced imaging capabilities may further expand the use of biodegradable scaffolds in tissue engineering. The main methods currently available are the blending of 2,3,5-triiodobenzoic acid (TIBA) (Singhana et al., 2015; Zhao et al., 2020), gold nanoparticles (AuNPs) (Tian et al., 2017; Tian et al., 2018; Huang et al., 2020) or bismuth nanoparticles (BiNPs) (Perez et al., 2021; Damasco et al., 2022) into PPDO to increase its imaging capacity.

Completely biodegradable IVCFs degrade completely after functioning and have less impact on the human body than partially biodegradable filters because they leave no residual material in the body and have a lower probability of related complications, but at the same time, they place higher demands on the overall degradation time, degradation rate, control of the degradation method and the manufacturing process.

The available studies on biodegradable filters have shown good thrombus interception and good thrombus capture in vitro and animal studies. However, displacement of the filters, which was reported in several papers (Gao et al., 2011; Zhang et al., 2017), is noteworthy and may be due to the inferior nature of the organic polymer materials used in these filters in terms of mechanical strength, radial support and the degree of recovery from deformation after release compared to metallic materials. Most of the above studies have not studied the specific degradation time, degradation process, and degradation products and their destination and effects on the body, and the formation process, size, destination and effects of the degradation products may become a constraint to the biodegradable filter in the clinic.

### 7.3 Biodegradable metal material

The most common materials for making biodegradable vena cava filters are organic polymers, such as polycaprolactone, PLLA, and PPDO. When compared to metallic materials, the radial force and mechanical strength of these organic polymer compounds, as well as their ability to recover from deformation after release, are weak; most of them cannot be fully expanded by the balloon and require additional energy, which is potentially dangerous for the vessel wall. Currently, biodegradable metals have demonstrated good performance in the health care field, with a variety of cardiovascular stents made from biodegradable metals emerging, demonstrating the good biocompatibility of biodegradable metals in vasculature (Bowen et al., 2016; Debieux et al., 2016; Shreenivas and Kereiakes, 2018), and biodegradable metals show significant promise in the direction of making biodegradable vena cava filters. Over the last few decades, biodegradable metals (BMs) as temporary implants have generated much scholarly interest in research.

BMs are metals expected to corrode gradually *in vivo*, with an appropriate host response elicited by the released corrosion products, which can pass through or be metabolized or assimilated by cells and/or tissue and then dissolve completely upon fulfilling the mission to assist with tissue healing with no implant residues (Liu et al., 2019). This is also known as an absorbable metal material.

In contrast to conventional metallic biomaterials, BMs consist mainly of essential metallic elements that are present in trace amounts in the human body. To date, there are three main BM systems that have been extensively studied, namely, Mg-based BM, Fe-based BM and Zn-based BM. For decades, Mg-based and Fe-based metals and zinc alloys have been a popular topic of research in the field of BMs (Yuan et al., 2022).

Although Fe-based BMs have excellent biocompatibility, corrosion resistance and mechanical properties, Fe-based alloys undergo degradation *in vivo* rather slowly without systemic iron toxicity, so implants can persist *in vivo* for a long time (Kraus et al., 2014). However, the corrosion products of Fe are stable in the organism and accumulate over time in the body, compromising the integrity of the vessel wall and causing damage to it, making this metal unavailable for the fabrication of absorbable implants that require a large volume or cross-sectional thickness (Peuster et al., 2006; Zhang et al., 2010; Pierson et al., 2012).

Existing BM research on zinc-based alloys, magnesium-based alloys, and zinc-magnesium alloys has revealed potentially outstanding qualities as material choices for biodegradable vena cava filters.

## 8 Summary

This article reviews the use of IVCFs in orthopaedics, the current status of filters and the progress of research into biodegradable vena cava filters and suggests possible future developments based on the published literature. With the long-term use of IVCFs, the associated difficulties are receiving increasing attention, and IVCF perfection is increasingly desired. Although there are still certain issues with current biodegradable filters, their ability to gradually dissolve and absorb in the body without the need for further surgery, as well as their ability to reduce the occurrence of numerous complications, will continue to be of concern to people. It is believed that with further development and perfection of the biodegradable filter through improvements in the manufacturing process and design concept, it will eventually be suitable for clinical use and may even be further developed into a drug-eluting biodegradable IVCF in the future, which will have increasingly broad application prospects.

## References

[B1] Aggarwal S. Patel S. Vashisht S. Kumar V. Sehgal I. S. Chauhan R. (2020). Guidelines for the prevention of venous thromboembolism in hospitalized patients with pelvi-acetabular trauma. J. Clin. Orthop. Trauma 11, 1002–1008. 10.1016/j.jcot.2020.09.011 33192002 PMC7656470

[B2] Agko M. Nazzal M. Jamil T. Castillo-Sang M. Clark P. Kasper G. (2010). Prevention of cardiopulmonary embolization of polymethylmethacrylate c ement fragment after kyphoplasty with insertion of inferior vena cava filter. J. Vasc. Surg. 51, 210–213. 10.1016/j.jvs.2009.07.110 19837540

[B3] Ahmed O. Madassery S. Heussner D. Tran P. Masrani A. Arslan B. (2016). VenaTech LP filter retrieval for filter-related thrombosis or malpositioning. J. Vasc. Interv. Radiol. 27, 1724–1726. 10.1016/j.jvir.2016.06.006 27926397

[B4] Al-Hakim R. Kee S. T. Olinger K. Lee E. W. Moriarty J. M. McWilliams J. P. (2014). Inferior vena cava filter retrieval: Effectiveness and complications o f routine and advanced techniques. J. Vasc. Interv. Radiol. 25, 933–939. 10.1016/j.jvir.2014.01.019 24630748

[B5] Alvarado A. M. Porto G. B. F. Wessell J. Buchholz A. L. Arnold P. M. (2020). Venous thromboprophylaxis in spine surgery. Glob. Spine J. 10, 65s–70s. 10.1177/2192568219858307 PMC6947674 31934524

[B6] Athreya S. Mathias N. Rogers P. Edwards R. (2009). Retrieval of cement embolus from inferior vena cava after percutaneous vertebroplasty. Cardiovasc. Interv. Radiol. 32, 817–819. 10.1007/s00270-009-9550-6 19333651

[B7] Austin M. S. Parvizi J. Grossman S. Restrepo C. Klein G. R. Rothman R. H. (2007). The inferior vena cava filter is effective in preventing fatal pulmonary embolus after hip and knee arthroplasties. J. Arthroplasty 22, 343–348. 10.1016/j.arth.2006.10.008 17400088

[B8] Awad J. N. Kebaish K. M. Donigan J. Cohen D. B. Kostuik J. P. (2005). Analysis of the risk factors for the development of post-operative spinal epidural haematoma. J. Bone Jt. Surg. Br. volume 87, 1248–1252. 10.1302/0301-620x.87b9.16518 16129751

[B9] Bauer K. A. Hawkins D. W. Peters P. C. Petitou M. Herbert J. M. Boeckel C. A. A. (2002). Fondaparinux, a synthetic pentasaccharide: The first in a new class of antithrombotic agents - the selective factor Xa inhibitors. Cardiovasc. Drug Rev. 20, 37–52. 10.1111/j.1527-3466.2002.tb00081.x 12070533

[B10] Baumann A. Tauss J. Baumann G. Tomka M. Hessinger M. Tiesenhausen K. (2006). Cement embolization into the vena cava and pulmonal arteries after ver tebroplasty: Interdisciplinary management. Eur. J. Vasc. Endovasc. Surg. 31, 558–561. 10.1016/j.ejvs.2005.11.008 16376118

[B11] Bicalho P. S. Hozack W. J. Rothman R. H. Eng K. (1996). Treatment of early symptomatic pulmonary embolism after total joint arthroplasty. J. Arthroplasty 11, 522–524. 10.1016/s0883-5403(96)80103-3 8872569

[B12] Bjørnarå B. T. Gudmundsen T. E. Dahl O. E. (2006). Frequency and timing of clinical venous thromboembolism after major jo int surgery. J. Bone Jt. Surg. Br. volume 88, 386–391. 10.1302/0301-620X.88B3.17207 16498018

[B13] Bovyn G. Gory P. Reynaud P. Ricco J. B. (1997). The tempofilter: A multicenter study of a new temporary caval filter implantable for up to six weeks. Ann. Vasc. Surg. 11, 520–528. 10.1007/s100169900084 9302065

[B14] Bowen P. K. Shearier E. R. Zhao S. Guillory R. J. Zhao F. Goldman J. (2016). Biodegradable metals for cardiovascular stents: From clinical concerns to recent Zn-alloys. Adv. Healthc. Mat. 5, 1121–1140. 10.1002/adhm.201501019 PMC4904226 27094868

[B15] Caplin D. M. Nikolic B. Kalva S. P. Ganguli S. Saad W. E. Zuckerman D. A. (2011). Quality improvement guidelines for the performance of inferior vena ca va filter placement for the prevention of pulmonary embolism. J. Vasc. Interv. Radiol. 22, 1499–1506. 10.1016/j.jvir.2011.07.012 21890380

[B16] Cloney M. B. Yamaguchi J. T. Dhillon E. S. Hopkins B. Smith Z. A. Koski T. R. (2018). Venous thromboembolism events following spinal fractures: A single center experience. Clin. Neurol. Neurosurg. 174, 7–12. 10.1016/j.clineuro.2018.08.030 30189328

[B17] Cloney M. B. Driscoll C. B. Yamaguchi J. T. Hopkins B. Dahdaleh N. S. (2020). Comparison of inpatient versus post-discharge venous thromboembolic events after spinal surgery: A single institution series of 6869 consecutive patients. Clin. Neurol. Neurosurg. 196, 105982. 10.1016/j.clineuro.2020.105982 32570019

[B18] Cohen-Levy W. B. Liu J. Sen M. Teperman S. H. Stone M. E. Jr. (2019). Prophylactic inferior vena cava filters for operative pelvic fractures: A twelve year experience. Int. Orthop. 43, 2831–2838. 10.1007/s00264-019-04384-0 31392493

[B19] Crochet D. P. Stora O. Ferry D. Grossetete R. Leurent B. Brunel P. (1993). Vena tech-LGM filter: Long-term results of a prospective study. Radiology 188, 857–860. 10.1148/radiology.188.3.8351362 8351362

[B20] Crochet D. P. Brunel P. Trogrlic S. Grossetete R. Auget J. L. Dary C. (1999). Long-term follow-up of vena tech-LGM filter: Predictors and frequency of caval occlusion. J. Vasc. Interv. Radiol. 10, 137–142. 10.1016/s1051-0443(99)70455-0 10082099

[B21] Dahl O. E. Colwell C. Frostick S. Haas S. Hull R. Laporte S. (2005). Fatal vascular outcomes following major orthopedic surgery. Thromb. Haemost. 93, 860–866. 10.1160/TH04-11-0769 15886800

[B22] Dake M. D. Murphy T. P. Kramer A. H. Darcy M. D. Sewall L. E. Curi M. A. (2018). One-year analysis of the prospective multicenter SENTRY clinical trial: Safety and effectiveness of the novate Sentry bioconvertible inferior vena cava filter. J. Vasc. Interv. Radiol. 29, 1350–1361. 10.1016/j.jvir.2018.05.009 30177423

[B23] Dake M. D. Murphy T. P. Kramer A. H. Darcy M. D. Sewall L. E. Curi M. A. (2020). Final two-year outcomes for the Sentry bioconvertible inferior vena cava filter in patients requiring temporary protection from pulmonary embolism. J. Vasc. Interv. Radiol. 31, 221–230. 10.1016/j.jvir.2019.08.036 31711748

[B24] Damasco J. A. Huang S. Y. Perez J. V. D. Manongdo J. A. T. Dixon K. A. Williams M. L. (2022). Bismuth nanoparticle and polyhydroxybutyrate coatings enhance the radiopacity of absorbable inferior vena cava filters for fluoroscopy-guided placement and longitudinal computed tomography monitoring in pigs. ACS Biomater. Sci. Eng. 8, 1676–1685. 10.1021/acsbiomaterials.1c01449 35343679 PMC9045416

[B25] Dazley J. M. Wain R. Vellinga R. M. Cohen B. Agulnick M. A. (2012). Prophylactic inferior vena cava filters prevent pulmonary embolisms in high-risk patients undergoing major spinal surgery. J. Spinal Disord. Tech. 25, 190–195. 10.1097/BSD.0b013e31821532bd 21423052

[B26] De Oliveira Leite T. F. Silva T. O. E. Pereira O. I. Carnevale F. C. (2020). Retrievable celect™ filter placement in the superior vena cava: A case report. Int. J. Surg. Case Rep. 73, 105–108. 10.1016/j.ijscr.2020.06.100 32652249 PMC7352047

[B27] Debieux P. Franciozi C. E. Lenza M. Tamaoki M. J. Magnussen R. A. Faloppa F. (2016). Bioabsorbable versus metallic interference screws for graft fixation in anterior cruciate ligament reconstruction. Cochrane Database Syst. Rev. 7, Cd009772. 10.1002/14651858.CD009772.pub2 27450741 PMC6458013

[B28] Dorr L. D. Gendelman V. Maheshwari A. V. Boutary M. Wan Z. Long W. T. (2007). Multimodal thromboprophylaxis for total hip and knee arthroplasty based on risk assessment. J. Bone Jt. Surgery-American Volume 89, 2648–2657. 10.2106/jbjs.F.00235 18056497

[B29] Dria S. J. Eggers M. D. (2016). *In vitro* evaluation of clot capture efficiency of an absorbable vena cava filter. J. Vasc. Surg. Venous Lymphatic Disord. 4, 472–478. 10.1016/j.jvsv.2016.05.006 PMC5027067 27639002

[B30] Dupont P. A. (1976). The Mobin-Uddin umbrella filter in the management of proven and threatened pulmonary embolism. Ann. R. Coll. Surg. Engl. 58, 318–321. 942169 PMC2493728

[B31] Edwards C. 2nd Blight A. Kim K. Edwards C. (2015). Polymethylmethacrylate entrapped in inferior vena cava filter after a scheuermann kyphosis revision surgery. Spine Deform. 3, 604–607. 10.1016/j.jspd.2015.03.004 27927563

[B32] Eggers M. D. Reitman C. A. (2012). *In vitro* analysis of polymer candidates for the development of absorbable vascular filters. J. Vasc. Interv. Radiol. 23, 1023–1030. 10.1016/j.jvir.2012.05.039 22840802

[B33] Eggers M. D. McArthur M. J. Figueira T. A. Abdelsalam M. E. Dixon K. P. Pageon L. R. (2015). Pilot *in vivo* study of an absorbable polydioxanone vena cava filter. J. Vasc. Surg. Venous Lymphatic Disord. 3, 409–420. 10.1016/j.jvsv.2015.03.004 26992619

[B34] Eggers M. Rousselle S. Urtz M. Albright R. Will A. Jourden B. (2019). Randomized controlled study of an absorbable vena cava filter in a porcine model. J. Vasc. Interv. Radiol. 30, 1487–1494. 10.1016/j.jvir.2019.03.010 31202677 PMC6736596

[B35] El-Daly I. Reidy J. Culpan P. Bates P. (2013). Thromboprophylaxis in patients with pelvic and acetabular fractures: A short review and recommendations. Injury 44, 1710–1720. 10.1016/j.injury.2013.04.030 23816168

[B36] Elizondo G. Eggers M. Falcon M. Trevino M. Marrufo R. Perez C. (2020). First-in-Human study with eight patients using an absorbable vena cava filter for the prevention of pulmonary embolism. J. Vasc. Interv. Radiol. 31, 1817–1824. 10.1016/j.jvir.2020.07.021 33008719

[B37] Falck-Ytter Y. Francis C. W. Johanson N. A. Curley C. Dahl O. E. Schulman S. (2012). Prevention of VTE in orthopedic surgery patients: Antithrombotic thera py and prevention of thrombosis, 9th ed: American college of chest phy sicians evidence-based clinical practice guidelines. Chest 141, e278S–e325S. 10.1378/chest.11-2404 22315265 PMC3278063

[B38] Franchini M. (2005). Heparin-induced thrombocytopenia: An update. Thromb. J. 3, 14. 10.1186/1477-9560-3-14 16202170 PMC1262784

[B39] Fullen W. D. Miller E. H. Steele W. F. McDonough J. J. (1973). Prophylactic vena caval interruption in hip fractures. J. Trauma Inj. Infect. Crit. Care 13, 403–410. 10.1097/00005373-197305000-00001 4540786

[B40] Gaines P. A. Kolodgie F. D. Crowley G. Horan S. MacDonagh M. McLucas E. (2018). Sentry bioconvertible inferior vena cava filter: Study of stages of incorporation in an experimental ovine model. Int. J. Vasc. Med. 2018, 1–10. 10.1155/2018/6981505 PMC6077616 30112213

[B41] Gao X. Zhang J. Chen B. Yu H. Li J. Zhang S. (2011). A new self-convertible inferior vena cava filter: Experimental *in-vitro* and *in-vivo* evaluation. J. Vasc. Interv. Radiol. 22, 829–834. 10.1016/j.jvir.2011.02.018 21530312

[B42] Zhao J. M. He M. L. Xiao Z. M. Li T. S. Wu H. Jiang H. (2014). Different types of intermittent pneumatic compression devices for preventing venous thromboembolism in patients after total hip replacement. 10.1002/14651858.CD009543.pub3 PMC7100582 25528992

[B43] Geisbüsch P. Benenati J. F. Pena C. S. Couvillon J. Powell A. Gandhi R. (2012). Retrievable inferior vena cava filters: Factors that affect retrieval success. Cardiovasc. Interv. Radiol. 35, 1059–1065. 10.1007/s00270-011-0268-x 22042454

[B44] Gelbfish G. A. Ascer E. (1991). Intracardiac and intrapulmonary Greenfield filters: A long-term follow-up. J. Vasc. Surg. 14, a32195–a32617. 10.1067/mva.1991.32195 1942368

[B45] Gesell M. W. Gonzalez Della Valle A. Bartolome Garcia S. Memtsoudis S. G. Ma Y. Haas S. B. (2013). Safety and efficacy of multimodal thromboprophylaxis following total knee arthroplasty: A comparative study of preferential aspirin vs. routine coumadin chemoprophylaxis. J. Arthroplasty 28, 575–579. 10.1016/j.arth.2012.08.004 23142450

[B46] Glotzbecker M. P. Bono C. M. Wood K. B. Harris M. B. (1976). Postoperative spinal epidural hematoma: A systematic review. Spine 35, E413–E420. 10.1097/BRS.0b013e3181d9bb77 20431474

[B47] Godoy Monzon D. Iserson K. V. Cid A. Vazquez J. A. (2012). Oral thromboprophylaxis in pelvic trauma: A standardized protocol. J. Emerg. Med. 43, 612–617. 10.1016/j.jemermed.2011.09.006 22244290

[B48] Grewal S. Chamarthy M. R. Kalva S. P. (2016). Complications of inferior vena cava filters. Cardiovasc. Diagn. Ther. 6, 632–641. 10.21037/cdt.2016.09.08 28123983 PMC5220210

[B49] Günther R. W. Neuerburg J. Mossdorf A. Pfeffer J. Hoj A. R. Molgaard-Nielsen A. (2005). New optional IVC filter for percutaneous retrieval--*in vitro* evaluatio n of embolus capturing efficiency. Rofo 177, 632–636. 10.1055/s-2005-858109 15871077

[B50] Guo W. Zheng Q. Li B. Shi X. Xiang D. Wang C. (2015). An experimental study to determine the role of inferior vena cava filter in preventing bone cement implantation syndrome. Iran. J. Radiol. 12, e14142. 10.5812/iranjradiol.14142v2 26557267 PMC4632131

[B51] Hahn D. (2015). Retrievable filter update: The Denali vena cava filter. Semin. Interv. Radiol. 32, 379–383. 10.1055/s-0035-1564812 PMC4640910 26622101

[B52] Han Y. Xu B. Fu G. Wang X. Xu K. Jin C. (2018). A randomized trial comparing the NeoVas sirolimus-eluting bioresorbable scaffold and metallic everolimus-eluting stents. JACC Cardiovasc. Interv. 11, 260–272. 10.1016/j.jcin.2017.09.037 29413240

[B53] Han X. Sheng Y. Wu J. Wang W. (2022). Unretrievable IVC filter due to cement intravasation. Cardiovasc Interv. Radiol. 45, 1048–1050. 10.1007/s00270-022-03122-1 35304615

[B54] Herbstreit F. Kühl H. Peters J. (1976). A cemented caval vein filter: Case report. Spine 31, E917–E919. 10.1097/01.brs.0000245824.63150.ef 17108822

[B55] Hirano S. Funatsu A. Nakamura S. Ikeda T. (2021). Clinical evaluation of retrievable inferior vena cava filters for the prevention of pulmonary thromboembolism. Heart Vessels 36, 1756–1764. 10.1007/s00380-021-01856-5 33856536

[B56] Hoppe H. Nutting C. W. Smouse H. R. Vesely T. M. Pohl C. Bettmann M. A. (2006). Günther Tulip filter retrievability multicenter study including CT follow-up: Final report. J. Vasc. Interv. Radiol. 17, 1017–1023. 10.1097/01.rvi.90000223689.49091.76 16778236

[B57] Huang S. Y. Eggers M. McArthur M. J. Dixon K. A. McWatters A. Dria S. (2017). Safety and efficacy of an absorbable filter in the inferior vena cava to prevent pulmonary embolism in swine. Radiology 285, 820–829. 10.1148/radiol.2017161880 28708470 PMC5708283

[B58] Huang S. Y. Damasco J. A. Tian L. Lu L. Perez J. V. D. Dixon K. A. (2020). *In vivo* performance of gold nanoparticle-loaded absorbable inferior vena cava filters in a swine model. Biomater. Sci. 8, 3966–3978. 10.1039/d0bm00414f 32558854 PMC7386069

[B59] Huang J. Dai X. Zhang X. Li J. Huang M. Liu C. (2021). Retrievable inferior vena cava filter to prevent pulmonary embolism in patients with fractures and deep venous thrombosis of lower extremities: A single-center experience. J. Int. Med. Res. 49, 030006052110065. 10.1177/03000605211006591 PMC8047091 33845601

[B60] Hui A. C. Heras-Palou C. Dunn I. Triffitt P. D. Crozier A. Imeson J. (1996). Graded compression stockings for prevention of deep-vein thrombosis after hip and knee replacement. J. Bone Jt. Surg. Br. volume 78, 550–554. 10.1302/0301-620x.78b4.0780550 8682818

[B61] Iliescu B. Haskal Z. J. (2012). Advanced techniques for removal of retrievable inferior vena cava filt ers. Cardiovasc. Interv. Radiol. 35, 741–750. 10.1007/s00270-011-0205-z 21674279

[B62] Inagaki E. Farber A. Eslami M. H. Siracuse J. J. Rybin D. V. Sarosiek S. (2016). Improving the retrieval rate of inferior vena cava filters with a mult idisciplinary team approach. J. Vasc. Surg. Venous Lymphatic Disord. 4, 276–282. 10.1016/j.jvsv.2015.11.002 PMC5531451 27318045

[B63] Isaak A. Takes M. Kingsmore D. Gürke L. (2018). Endovascular retrieval of intracaval cement: A fishing net technique. Cardiovasc. Interv. Radiol. 41, 1958–1961. 10.1007/s00270-018-2061-6 30128782

[B64] Iwanaga J. Rustagi T. Ishak B. Johal J. David G. Reina M. A. (2020). Venous drainage of lumbar vertebral bodies: Anatomic study with application to kyphoplasty, vertebroplasty, and pedicle screw complications. World Neurosurg. x. 137, e286–e290. 10.1016/j.wneu.2020.01.174 32014549

[B65] Janvier A. L. Hamdan H. Malas M. (2010). Bilateral renal vein thrombosis and subsequent acute renal failure due to IVC filter migration and thrombosis. Clin. Nephrol. 73, 408–412. 10.5414/cnp73408 20420804

[B66] Kalina M. Bartley M. Cipolle M. Tinkoff G. Stevenson S. Fulda G. (2012). Improved removal rates for retrievable inferior vena cava filters with the use of a 'filter registry. Am. Surg. 78, 94–97. 10.1177/000313481207800143 22273323

[B67] Kalva S. P. Marentis T. C. Yeddula K. Somarouthu B. Wicky S. Stecker M. S. (2011). Long-term safety and effectiveness of the "OptEase" vena cava filter. Cardiovasc. Interv. Radiol. 34, 331–337. 10.1007/s00270-010-9969-9 PMC7103092 20820780

[B68] Kanter B. Moser K. M. (1988). The Greenfield vena cava filter. Chest 93, 170–175. 10.1378/chest.93.1.170 3275527

[B69] Karunakar M. A. Shah S. N. Jerabek S. (2005). Body mass index as a predictor of complications after operative treatment of acetabular fractures. J. Bone Jt. Surg. 87, 1498–1502. 10.2106/jbjs.D.02258 15995116

[B70] Kaufman J. A. Barnes G. D. Chaer R. A. Cuschieri J. Eberhardt R. T. Johnson M. S. (2020). Society of interventional radiology clinical practice guideline for in ferior vena cava filters in the treatment of patients with venous thro mboembolic disease: Developed in collaboration with the American college of cardiology, American college of chest physicians, American colle ge of surgeons committee on trauma, American heart association, societ y for vascular surgery, and society for vascular medicine. J. Vasc. Interv. Radiol. 31, 1529–1544. 10.1016/j.jvir.2020.06.014 32919823

[B71] Kepler C. K. McKenzie J. Kreitz T. Vaccaro A. (2018). Venous thromboembolism prophylaxis in spine surgery. J. Am. Acad. Orthop. Surg. 26, 489–500. 10.5435/jaaos-d-17-00561 29870417

[B72] Kim Y.-J. Choi D. H. Ahn S. Sohn C. H. Seo D. W. Kim W. Y. (2016). Timing of pulmonary embolisms in femur fracture patients: Incidence an d outcomes. J. Trauma Acute Care Surg. 80, 952–956. 10.1097/TA.0000000000001014 26891161

[B73] Konstantinides S. V. Torbicki A. Agnelli G. Danchin N. Fitzmaurice D. Galie N. (2014). 2014 ESC Guidelines on the diagnosis and management of acute pulmonary embolism. Eur. Heart J. 35, 3033–3080. 10.1093/eurheartj/ehu283 25173341

[B74] Kraus T. Moszner F. Fischerauer S. Fiedler M. Martinelli E. Eichler J. (2014). Biodegradable Fe-based alloys for use in osteosynthesis: Outcome of an *in vivo* study after 52 weeks. Acta Biomater. 10, 3346–3353. 10.1016/j.actbio.2014.04.007 24732635

[B75] Langlois J. Nich C. Courpied J. P. Hamadouche M. (2013). An unreported cause of early postoperative dislocation following total hip revision: Massive intra-capsular oedema related to inferior vena cava filter thrombosis. Orthop. Traumatology Surg. Res. 99, 367–370. 10.1016/j.otsr.2012.10.015 23491681

[B76] Laratta J. L. Shillingford J. N. Lombardi J. M. Mueller J. D. Reddy H. Saifi C. (2017). Utilization of vertebroplasty and kyphoplasty procedures throughout th e United States over a recent decade: An analysis of the nationwide in patient sample. J. Spine Surg. 3, 364–370. 10.21037/jss.2017.08.02 29057344 PMC5637187

[B77] Leask R. L. Johnston K. W. Ojha M. (2004). Hemodynamic effects of clot entrapment in the TrapEase inferior vena cava filter. J. Vasc. Interv. Radiol. 15, 485–490. 10.1097/01.rvi.0000124941.58200.85 15126659

[B78] Leon L. Rodriguez H. Tawk R. G. Ondra S. L. Labropoulos N. Morasch M. D. (2005). The prophylactic use of inferior vena cava filters in patients undergoing high-risk spinal surgery. Ann. Vasc. Surg. 19, 442–447. 10.1007/s10016-005-0025-1 15864473

[B79] Lewis S. Glen J. Dawoud D. Dias S. Cobb J. Griffin X. L. (2019). Venous thromboembolism prophylaxis strategies for people undergoing elective total knee replacement: A systematic review and network meta-analysis. Lancet Haematol. 6, e530–e539. 10.1016/s2352-3026(19)30155-3 31444124

[B80] Li Z. Ni R.-f. Zhao X. Yang C. Li M.-m. (2013). Cement embolus trapped in the inferior vena cava filter during percuta neous vertebroplasty. Korean J. Radiol. 14, 451–454. 10.3348/kjr.2013.14.3.451 23690712 PMC3655299

[B81] Li X. Haddadin I. McLennan G. Farivar B. Staub D. Beck A. (2020). Inferior vena cava filter - comprehensive overview of current indications, techniques, complications and retrieval rates. Vasa 49, 449–462. 10.1024/0301-1526/a000887 32660360

[B82] Liu W. C. Do Y. S. Choo S. W. Kim D. I. Kim Y. W. Kim D. K. (2005). The mid-term efficacy and safety of a permanent nitinol IVC filter(TrapEase). Korean J. Radiol. 6, 110–116. 10.3348/kjr.2005.6.2.110 15968150 PMC2686418

[B83] Liu Y. Zheng Y. Chen X. Yang J. Pan H. Chen D. (2019). Fundamental theory of biodegradable metals-definition, criteria, and design. Adv. Funct. Mat. 29, 1805402. 10.1002/adfm.201805402

[B84] Marquess J. S. Burke C. T. Beecham A. H. Dixon R. G. Stavas J. M. Sag A. A. (2008). Factors associated with failed retrieval of the Günther Tulip inferior vena cava filter. J. Vasc. Interv. Radiol. 19, 1321–1327. 10.1016/j.jvir.2008.06.004 18725095

[B85] McClendon J. Jr. OʼShaughnessy B. A. Smith T. R. Sugrue P. A. Halpin R. J. Morasch M. (1976). Comprehensive assessment of prophylactic preoperative inferior vena cava filters for major spinal reconstruction in adults. Spine 37, 1122–1129. 10.1097/BRS.0b013e31824abde2 22281478

[B86] Minocha J. Idakoji I. Riaz A. Karp J. Gupta R. Chrisman H. B. (2010). Improving inferior vena cava filter retrieval rates: Impact of a dedic ated inferior vena cava filter clinic. J. Vasc. Interv. Radiol. 21, 1847–1851. 10.1016/j.jvir.2010.09.003 21035356

[B87] Mismetti P. Laporte S. Pellerin O. Ennezat P. V. Couturaud F. Elias A. (2015). Effect of a retrievable inferior vena cava filter plus anticoagulation vs anticoagulation alone on risk of recurrent pulmonary embolism: A r andomized clinical trial. JAMA 313, 1627–1635. 10.1001/jama.2015.3780 25919526

[B88] Montgomery K. D. Geerts W. H. Potter H. G. Helfet D. L. (1996). Thromboembolic complications in patients with pelvic trauma. Clin. Orthop. Relat. Res. 329, 68–87. 10.1097/00003086-199608000-00010 8769438

[B89] Moorthy V. Wang J. X. Tang J. H. Oh J. Y. (2020). Postoperative spinal epidural hematoma following therapeutic anticoagulation: Case report and review of literature. J. Spine Surg. 6, 743–749. 10.21037/jss-20-636 33447677 PMC7797790

[B90] Murphy E. H. Johnson E. D. Kopchok G. E. Fogarty T. J. Arko F. R. (2009). Crux vena cava filter. Expert Rev. Med. Devices 6, 477–485. 10.1586/erd.09.25 19751120

[B91] Nutescu E. A. Shorr A. F. Farrelly E. Horblyuk R. Happe L. E. Franklin M. (2008). Burden of deep vein thrombosis in the outpatient setting following maj or orthopedic surgery. Ann. Pharmacother. 42, 1216–1221. 10.1345/aph.1L135 18611992

[B92] Olson S. T. Chuang Y. J. (2002). Heparin activates antithrombin anticoagulant function by generating new interaction sites (exosites) for blood clotting proteinases. Trends cardiovasc. Med. 12, 331–338. 10.1016/s1050-1738(02)00183-4 12536119

[B93] Pan Y. Zhao J. Mei J. Shao M. Zhang J. Wu H. (2016). Evaluation of nonpermanent inferior vena cava filter placement in patients with deep venous thrombosis after lower extremity fracture: A single-center retrospective study. Phlebology. 31, 564–572. 10.1177/0268355515597632 26249151

[B94] Parvizi J. Huang R. Raphael I. J. Maltenfort M. G. Arnold W. V. Rothman R. H. (2015). Timing of symptomatic pulmonary embolism with warfarin following arthr oplasty. J. Arthroplasty 30, 1050–1053. 10.1016/j.arth.2015.01.004 25648058

[B95] Pellerin O. Barral F. G. Lions C. Novelli L. Beregi J. P. Sapoval M. (2008). Early and late retrieval of the ALN removable vena cava filter: Results from a multicenter study. Cardiovasc. Interv. Radiol. 31, 889–896. 10.1007/s00270-008-9357-x 18493821

[B96] Perez J. V. D. Jacobsen M. C. Damasco J. A. Melancon A. Huang S. Y. Layman R. R. (2021). Optimization of the differentiation and quantification of high-Z nanoparticles incorporated in medical devices for CT-guided interventions. Med. Phys. 48, 300–312. 10.1002/mp.14601 33216978 PMC8097741

[B97] Peuster M. Hesse C. Schloo T. Fink C. Beerbaum P. von Schnakenburg C. (2006). Long-term biocompatibility of a corrodible peripheral iron stent in the porcine descending aorta. Biomaterials 27, 4955–4962. 10.1016/j.biomaterials.2006.05.029 16765434

[B98] Pierson D. Edick J. Tauscher A. Pokorney E. Bowen P. Gelbaugh J. (2012). A simplified *in vivo* approach for evaluating the bioabsorbable behavior of candidate stent materials. J. Biomed. Mat. Res. 100, 58–67. 10.1002/jbm.b.31922 21905215

[B99] Ploumis A. Ponnappan R. K. Sarbello J. Dvorak M. Fehlings M. G. Baron E. (1976). Thromboprophylaxis in traumatic and elective spinal surgery: Analysis of questionnaire response and current practice of spine trauma surgeons. Spine 35, 323–329. 10.1097/BRS.0b013e3181ca652e 20075763

[B100] Poletti P. A. Becker C. D. Prina L. Ruijs P. Bounameaux H. Didier D. (1998). Long-term results of the Simon nitinol inferior vena cava filter. Eur. Radiol. 8, 289–294. 10.1007/s003300050382 9477285

[B101] Prater S. Awan M. A. Antuna K. Colon J. Z. (2021). Prevention of pulmonary cement embolism by inferior vena cava filter f ollowing vertebroplasty-related cement intravasation. J. Radiol. Case Rep. 15, 17–27. 10.3941/jrcr.v15i4.4139 PMC8253145 34276872

[B102] Prince M. R. Salzman E. W. Schoen F. J. Palestrant A. M. Simon M. (1988). Local intravascular effects of the nitinol wire blood clot filter. Invest. Radiol. 23, 294–300. 10.1097/00004424-198804000-00009 3286574

[B103] Raphael I. J. McKenzie J. C. Zmistowski B. Brown D. B. Parvizi J. Austin M. S. (2014). Pulmonary embolism after total joint arthroplasty: Cost and effectiveness of four treatment modalities. J. Arthroplasty 29, 933–937. 10.1016/j.arth.2013.09.033 24269095

[B104] Roehm J. O. F. (1984). The bird's nest filter: A new percutaneous transcatheter inferior vena cava filter. J. Vasc. Surg. 1, 498–501. 10.1016/0741-5214(84)90092-2 6481901

[B105] Rosner M. K. Kuklo T. R. Tawk R. Moquin R. Ondra S. L. (2004). Prophylactic placement of an inferior vena cava filter in high-risk patients undergoing spinal reconstruction. Neurosurg. Focus 17, 1–6. 10.3171/foc.2004.17.4.6 15633992

[B106] Schulman S. Majeed A. (2011). A benefit-risk assessment of dabigatran in the prevention of venous thromboembolism in orthopaedic surgery. Drug Saf. 34, 449–463. 10.2165/11587290-000000000-00000 21585219

[B107] Segon Y. S. Summey R. D. Slawski B. Kaatz S. (1995). Surgical venous thromboembolism prophylaxis: Clinical practice update. Hosp. Pract. (1995). 48, 248–257. 10.1080/21548331.2020.1788893 32589468

[B108] Shimoo S. Koide M. (2020). Incomplete opening of an ALN-type inferior vena cava filter due to entanglement of the filter legs resulting in filter migration and inferior vena cava perforation. Radiol. Case Rep. 15, 1231–1234. 10.1016/j.radcr.2020.05.019 32577139 PMC7300239

[B109] Shreenivas S. S. Kereiakes D. J. (2018). Evolution of the SYNERGY bioresorbable polymer metallic coronary stent. Future Cardiol. 14, 307–317. 10.2217/fca-2018-0040 29926758

[B110] Singer M. A. Wang S. L. (2011). Modeling blood flow in a tilted inferior vena cava filter: Does tilt a dversely affect hemodynamics? J. Vasc. Interv. Radiol. 22, 229–235. 10.1016/j.jvir.2010.09.032 21211992

[B111] Singhana B. Chen A. Slattery P. Yazdi I. K. Qiao Y. Tasciotti E. (2015). Infusion of iodine-based contrast agents into poly(p-dioxanone) as a radiopaque resorbable IVC filter. J. Mat. Sci. Mat. Med. 26, 124. 10.1007/s10856-015-5460-0 PMC6154385 25690619

[B112] Song G. Zhao H. Q. Liu Q. Fan Z. (2022). A review on biodegradable biliary stents: Materials and future trends. Bioact. Mat. 17, 488–495. 10.1016/j.bioactmat.2022.01.017 PMC8968460 35415292

[B113] Sostres C. Lanas A. (2011). Gastrointestinal effects of aspirin. Nat. Rev. Gastroenterol. Hepatol. 8, 385–394. 10.1038/nrgastro.2011.97 21647198

[B114] Spitalny A. D. (2006). Bioabsorbable implants. Clin. Podiatr. Med. Surg. 23, 673–694. 10.1016/j.cpm.2006.06.001 17067887

[B115] Stein P. D. Matta F. Hughes M. J. (2018). Prophylactic inferior vena cava filters in patients with fractures of the pelvis or long bones. J. Clin. Orthop. Trauma 9, 175–180. 10.1016/j.jcot.2017.09.018 29896024 PMC5995070

[B116] Stösslein F. Altmann E. (1998). A rare complication with an Anthéor vena cava filter. Cardiovasc. Interv. Radiol. 21, 165–167. 10.1007/s002709900235 9502686

[B117] Sutphin P. D. Reis S. P. McKune A. Ravanzo M. Kalva S. P. Pillai A. K. (2015). Improving inferior vena cava filter retrieval rates with the define, m easure, analyze, improve, control methodology. J. Vasc. Interv. Radiol. 26, 491–498. 10.1016/j.jvir.2014.11.030 25636673

[B118] Sweeney T. J. Van Aman M. E. (1993). Deployment problems with the titanium Greenfield filter. J. Vasc. Interv. Radiol. 4, 691–694. 10.1016/s1051-0443(93)71950-8 8219566

[B119] Talec P. Gaujoux S. Samama C. M. (2016). Early ambulation and prevention of post-operative thrombo-embolic risk. J. Visc. Surg. 153, S11–s14. 10.1016/j.jviscsurg.2016.09.002 27789264

[B120] Tapson V. F. Hazelton J. P. Myers J. Robertson C. Gilani R. Dunn J. A. (2017). Evaluation of a device combining an inferior vena cava filter and a central venous catheter for preventing pulmonary embolism among critically ill trauma patients. J. Vasc. Interv. Radiol. 28, 1248–1254. 10.1016/j.jvir.2017.05.001 28642012

[B121] Thors A. Muck P. (2011). Resorbable inferior vena cava filters: Trial in an *in-vivo* porcine model. J. Vasc. Interv. Radiol. 22, 330–335. 10.1016/j.jvir.2010.11.030 21353986

[B122] Tian L. Lee P. Singhana B. Chen A. Qiao Y. Lu L. (2017). Radiopaque resorbable inferior vena cava filter infused with gold nanoparticles. Sci. Rep. 7, 2147. 10.1038/s41598-017-02508-3 28526874 PMC5438341

[B123] Tian L. Lee P. Singhana B. Chen A. Qiao Y. Lu L. (2018). *In vivo* imaging of radiopaque resorbable inferior vena cava filter infused with gold nanoparticles. Proc. SPIE. Int. Soc. Opt. Eng. 10576, 105762S. 10.1117/12.2293738 31406393 PMC6690051

[B124] Toro J. B. Gardner M. J. Hierholzer C. Sama D. Kosi C. Ertl W. (2008). Long-term consequences of pelvic trauma patients with thromboembolic disease treated with inferior vena caval filters. J. Trauma 65, 25–29. 10.1097/TA.0b013e318075e97a 18580529

[B125] Uberoi R. Tapping C. R. Chalmers N. Allgar V. (2013). British society of interventional Radiology (BSIR) inferior vena cava (IVC) filter registry. Cardiovasc. Interv. Radiol. 36, 1548–1561. 10.1007/s00270-013-0606-2 23511992

[B126] Vaishya R. Chauhan M. Vaish A. (2013). Bone cement. J. Clin. Orthop. Trauma 4, 157–163. 10.1016/j.jcot.2013.11.005 26403875 PMC3880950

[B127] Weinberg I. Kaufman J. Jaff M. R. (2013). Inferior vena cava filters. JACC Cardiovasc. Interv. 6, 539–547. 10.1016/j.jcin.2013.03.006 23787230

[B128] Weitz J. I. (1997). Low-molecular-weight heparins. N. Engl. J. Med. Overseas. Ed. 337, 688–698. 10.1056/nejm199709043371007 9278467

[B129] Witt D. M. Clark N. P. Kaatz S. Schnurr T. Ansell J. E. (2016). Guidance for the practical management of warfarin therapy in the treatment of venous thromboembolism. J. Thromb. Thrombolysis 41, 187–205. 10.1007/s11239-015-1319-y 26780746 PMC4715850

[B130] Yakkanti R. R. Mohile N. V. Cohen-Levy W. B. Haziza S. Lavelle M. J. Bellam K. G. (2021). Perioperative management of acetabular and pelvic fractures: Evidence-based recommendations. Arch. Orthop. Trauma Surg. 10.1007/s00402-021-04278-0 34854977

[B131] Yang C. Ma F. Gao C. Kang Y. Zhang G. Liu P. (2019). Design and evaluation of a novel biodegradable inferior vena cava filter. J. Biomater. Appl. 33, 1060–1069. 10.1177/0885328218824203 30862277

[B132] Yin T. Du R. Wang Y. Huang J. Huang Y. (2022). Two-stage degradation and novel functional endothelium characteristics of a 3-D printed bioresorbable scaffold. Bioact. Mat. 10, 378–396. 10.1016/j.bioactmat.2021.08.020 PMC8636822 34901554

[B133] Yoo K. Y. Jeong S. W. Yoon W. Lee J. (1976). Acute respiratory distress syndrome associated with pulmonary cement e mbolism following percutaneous vertebroplasty with polymethylmethacryl ate. Spine 29, E294–E297. 10.1097/01.brs.0000131211.87594.b0 15247590

[B134] Yuan W. Xia D. Wu S. Zheng Y. Guan Z. Rau J. V. (2022). A review on current research status of the surface modification of Zn-based biodegradable metals. Bioact. Mat. 7, 192–216. 10.1016/j.bioactmat.2021.05.018 PMC8379348 34466727

[B135] Zhang E. Chen H. Shen F. (2010). Biocorrosion properties and blood and cell compatibility of pure iron as a biodegradable biomaterial. J. Mat. Sci. Mat. Med. 21, 2151–2163. 10.1007/s10856-010-4070-0 20396936

[B136] Zhang F. Li H. Liang G. Zhang H. (2017). Development and evaluation of a new biodegradable vena cava filter in a canine model. Asian J. Surg. 40, 12–16. 10.1016/j.asjsur.2015.05.002 26216258

[B137] Zhao F. Xu H. Xue W. Li Y. Sun J. Wang F. (2020). Iodinated poly(p-dioxanone) as a facile platform for X-ray imaging of resorbable implantable medical devices. J. Biomater. Appl. 35, 39–48. 10.1177/0885328220912842 32192387

