# Supplementary material for: The application of inferior vena cava filters in orthopaedics and current research advances
Source: Front Bioeng Biotechnol. 2022 Nov 21;10:1045220. doi: 10.3389/fbioe.2022.1045220 (PMC9719953; doi:10.3389/fbioe.2022.1045220)
Supplement: Supplementary file 1 [file DataSheet1.docx]

Table:

Table 1:

| List of abbreviated words | |
| --- | --- |
| Inferior vena cava filters | IVCF |
| Deep vein thrombosis | DVT |
| Venous thromboembolism | VTE |
| Pulmonary embolism | PE |
| Inferior vena cava | IVC |
| Total knee arthroplasty | TKA |
| Total hip arthroplasty | THA |
| Low molecular weight heparin | LMWH |
| Unfractionated heparin | UFH |
| Total joint arthroplasty | TJA |
| Major orthopaedic surgery | MOS |
| Polymethyl methacrylate | PMMA |
| Percutaneous vertebroplasty | PVP |
| Standard retrieval | SR |
| Advanced endovascular retrieval | AER |
| Poly-l-actide | PLLA |
| Poly-l-actide vascular scaffolds | PLS |
| Poly(p-dioxanone) | PPDO |
| 2,3,5-triiodobenzoic acid | TIBA |
| Gold nanoparticles | AuNPs |
| Bismuth nanoparticles | BiNPs |
| Biodegradable metals | BM |

Table 2:

| Summary of ICVF interception of migrating bone cement | | | | |
| --- | --- | --- | --- | --- |
| Case | Filter implantation time | Filter type | Is the filter recycled | Recycling methods |
| 2006 | Pre-operative | Recyclable | Yes | Open surgery |
| 2009 | Post-operative | Recyclable | Yes | Interventional surgery |
| 2010 | Post-operative | Recyclable | Yes | Interventional surgery |
| 2013 | Pre-operative | Permanent | No | \ |
| 2015 | Pre-operative | Recyclable | Yes | Interventional surgery |
| 2021 | Pre-operative | Recyclable | No | \ |
| 2022 | Pre-operative | Recyclable | No | \ |

Figure:

Figure 1:

| 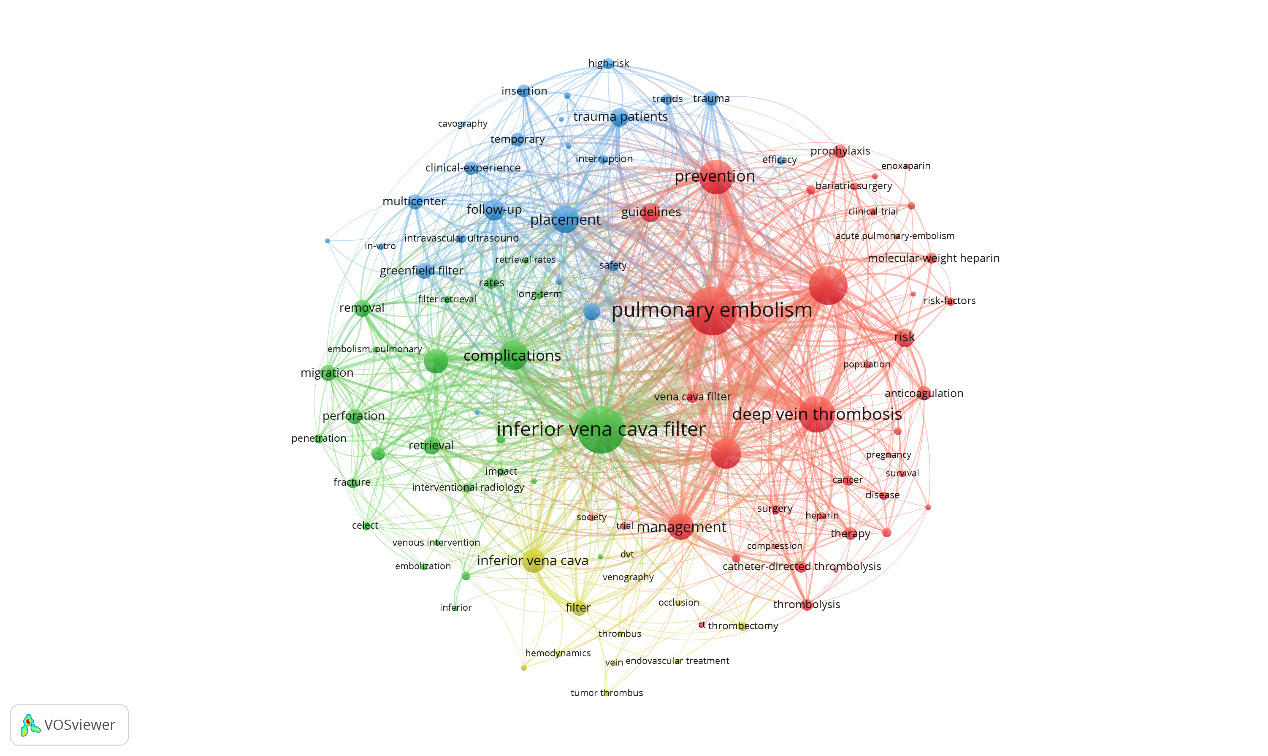 |
| --- |
| A comprehensive search of the Web of Science for literature related to vena cava filters was conducted, and the search results were imported into vosviewer and colored by the software for keyword relevance clustering analysis in the field. |

Figure 2:

| 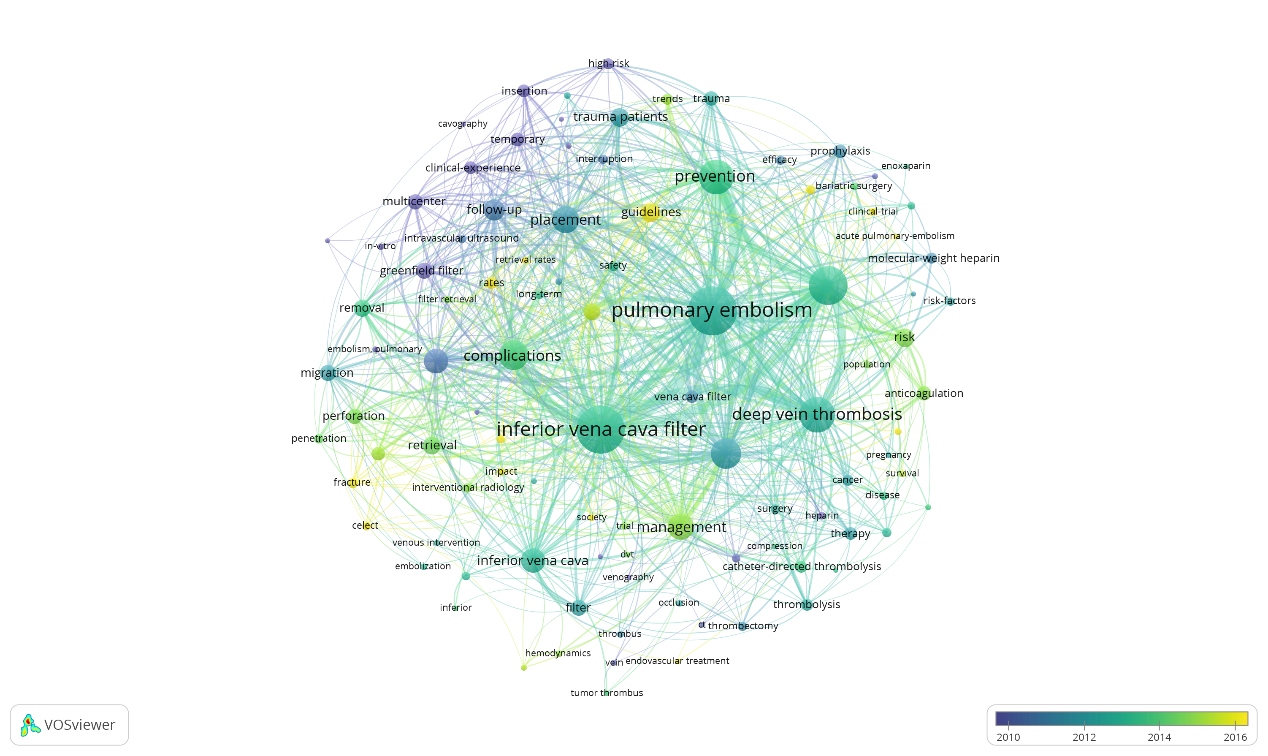 |
| --- |
| Then superimposing the time factor on Figure 1 we can obtain the average year of keyword appearance in the literature. |

Figure 3:

| 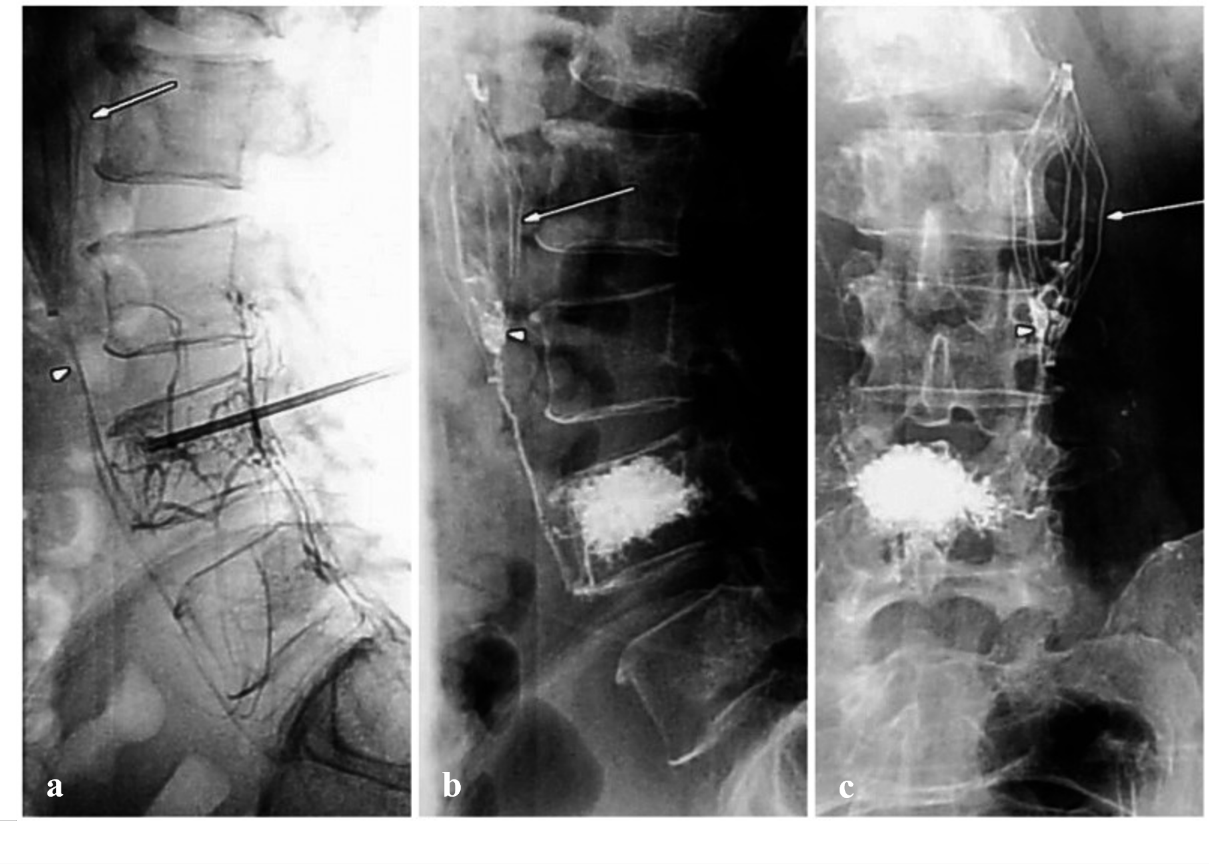 |
| --- |
| a: Cemented basilar venous drainage to the lateral vertebral plexus and IVC. Cement injection of L4 vertebrae: cement migrates into IVC and gets caught in filter.  b: Lateral fluoroscopy.  c: Anteroposterior fluoroscopy.  Image sourced from Li ^1^ |

Figure 4:

| 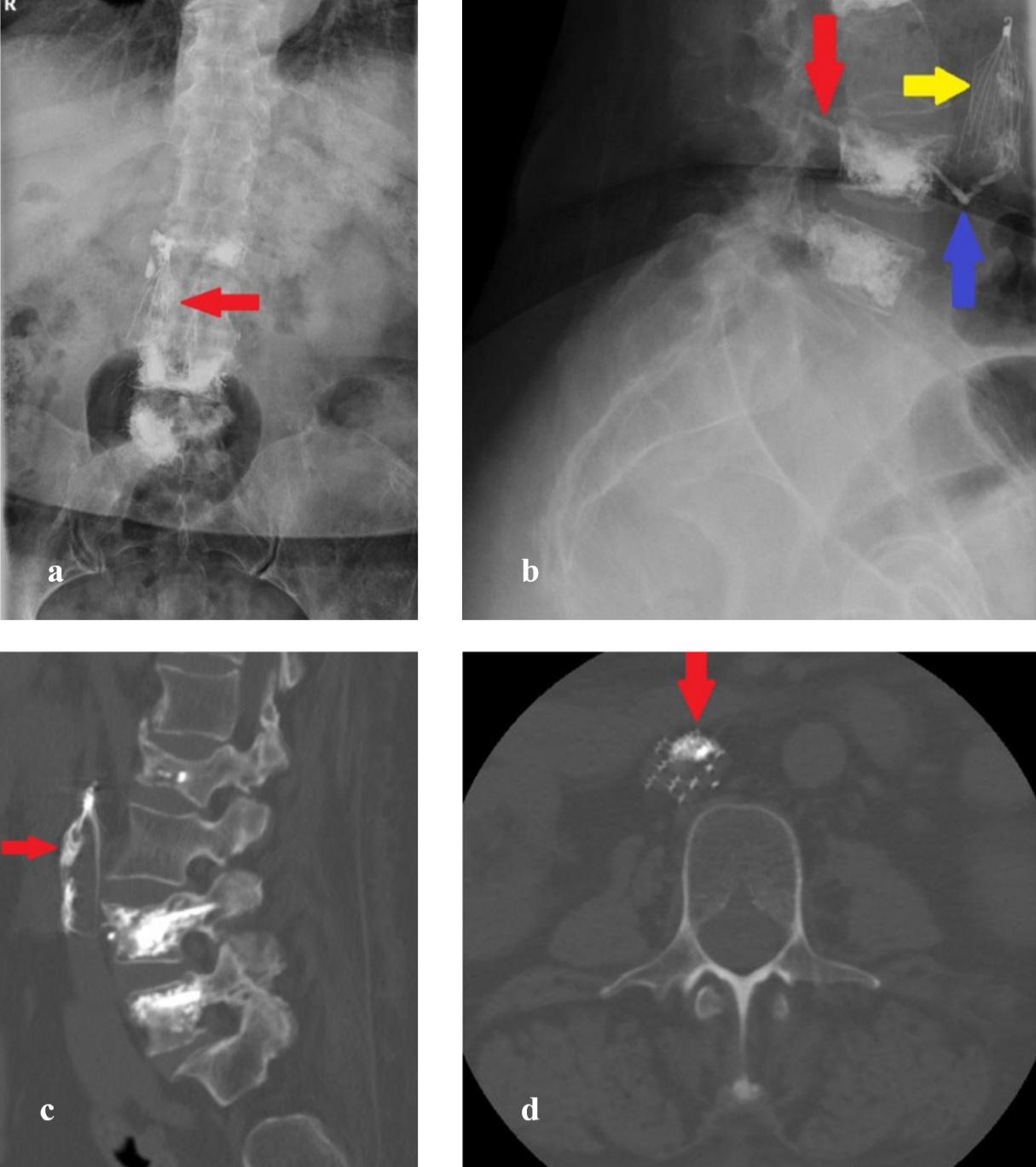 |
| --- |
| a: Vertebroplasty-related intravasation of bone cement into the inferior vena cava via small bridging veins of the anterior external vertebral venous plexus at the L4 level with subsequent entrapment by an IVCF (red arrow).  b: Lateral radiograph of the lumbar spine status after L2, L4 and L5 vertebroplasty. Linear opacities representing intravasated bone cement are seen extending dorsally along the catheter track (red arrow) and ventrally along the anterior external vertebral venous plexus draining into the inferior vena cava (blue arrow). Cement is seen within the spokes of an adjacent IVC filter (yellow arrow).  c: Axial noncontrast CT of the lumbar spine at the level of L3 demonstrates a piece of intravasated bone cement within the spokes of an IVC filter (red arrow).  d: Sagittal noncontrast CT of the lumbar spine status after L2, L4 and L5 vertebroplasty. Intravasated cement is seen within the spokes of an adjacent IVC filter (red arrow).  Image sourced from Prater ^2^ |

Figure 5:

| 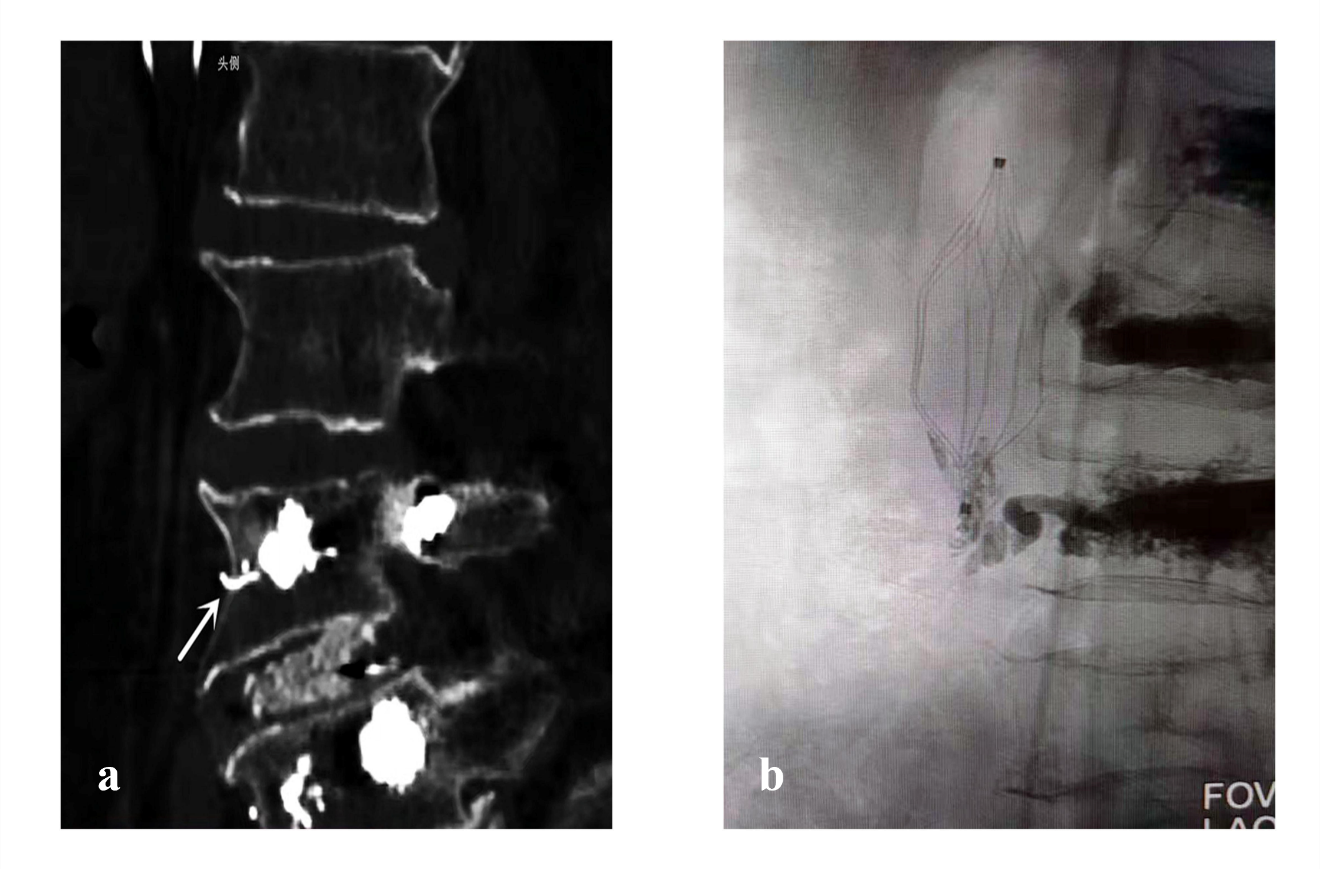 |
| --- |
| a:  CT scans revealed strip-like high density cement extravasation (arrows) in vertebra bone.  B: Venography showed cement attached to the IVC filter hook and trapped in the filter.  Image sourced from Han^3^ |

Figure 6:

| 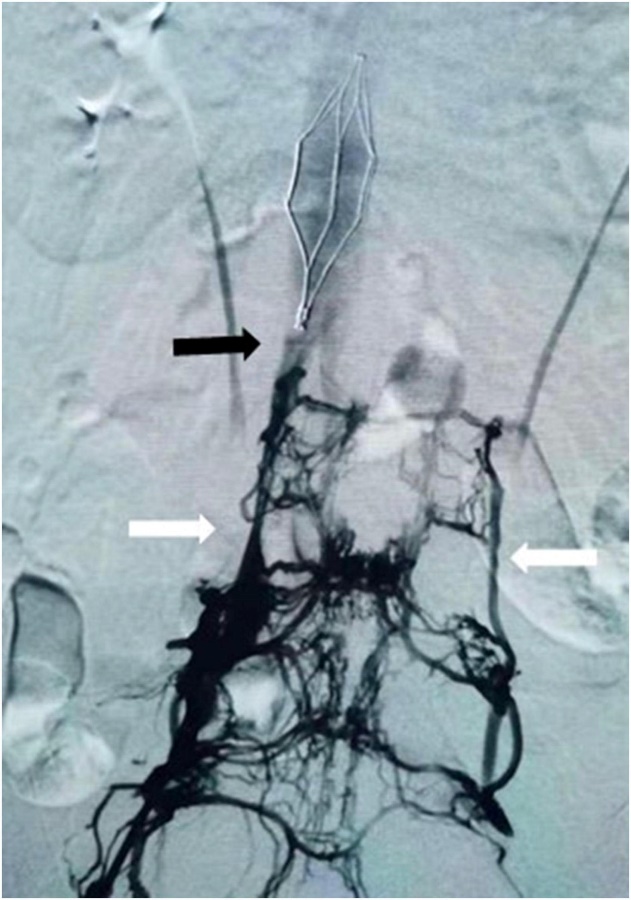 |
| --- |
| Venography showed collateral drainage veins between inferior vena cava (black arrow) and paravertebral venous (white arrow).  Image sourced from Han^3^ |

Figure 7:

| 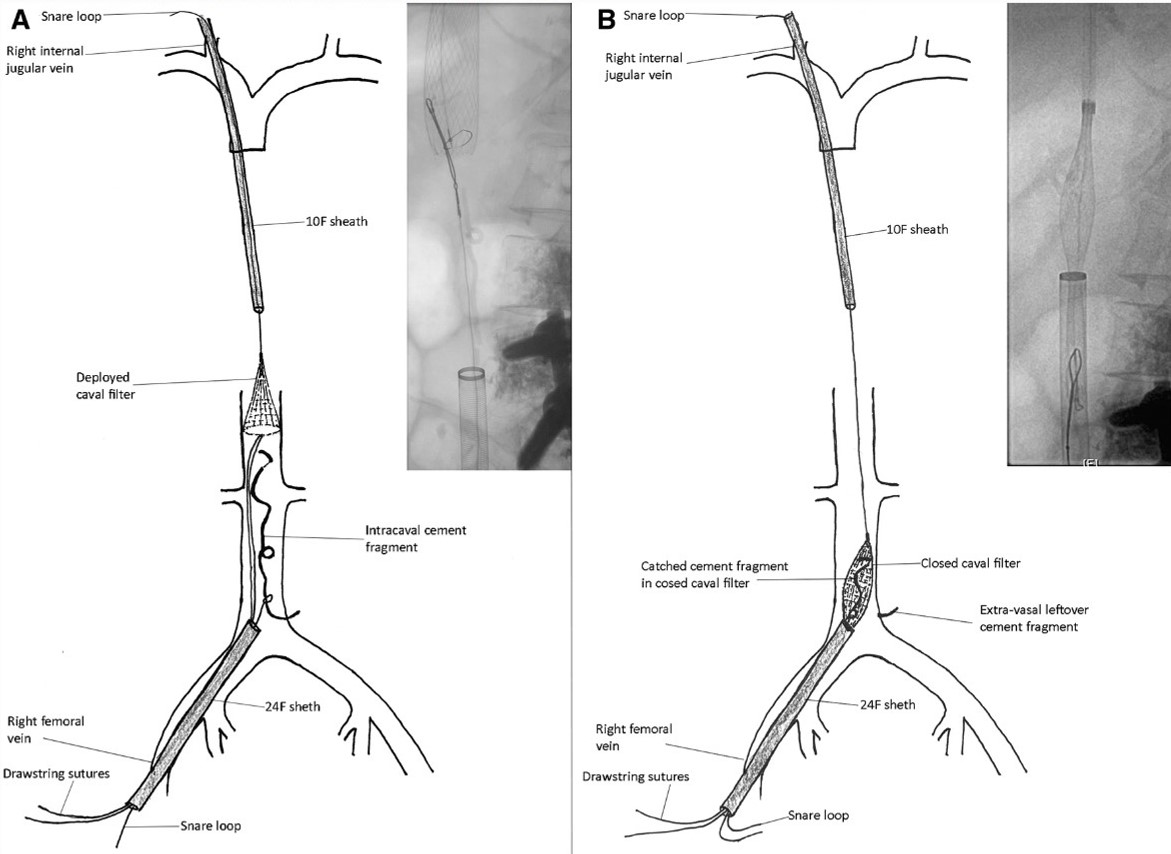 |
| --- |
| Schematic illustration and interventional imaging of the fishing net technique.  A: Open caval filter and snared cement fragment.  B: Retrieval of closed caval filter.  Image sourced from Isaak ^4^ |

Figure 8:

| 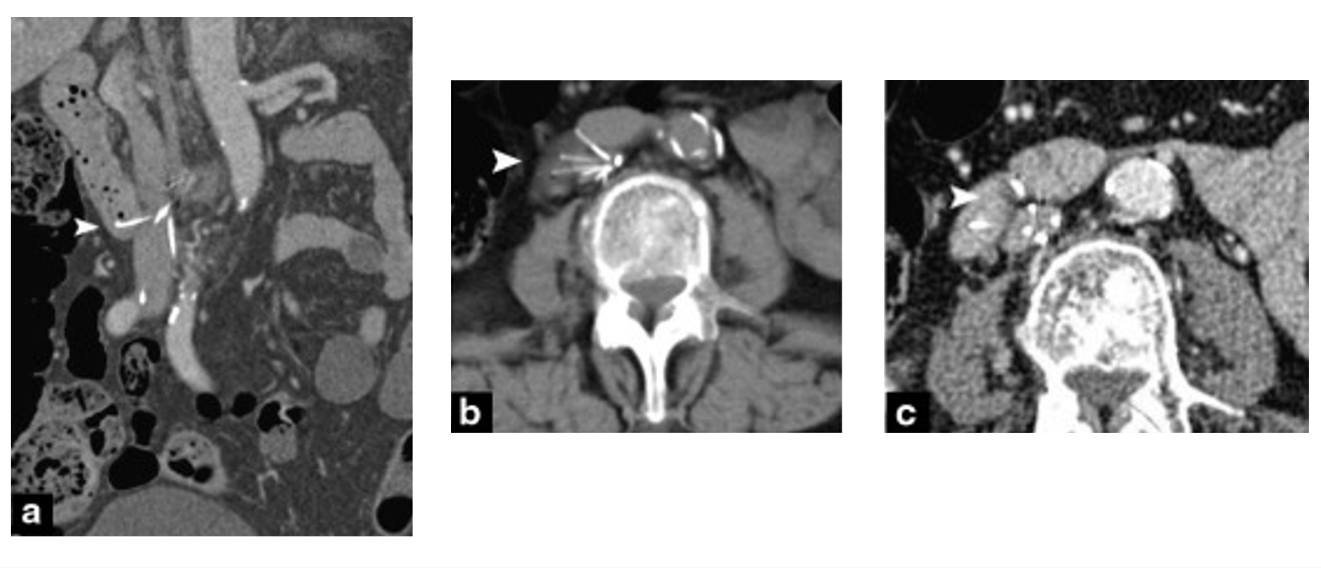 |
| --- |
| Abdominopelvic CT scan during the venous phase. MIP reconstructed image in the coronal plane (a) and in the axial plane (b). (c) Duodenal perforation due to IVC filter (arrowhead).  Image sourced from Bardin ^5^ |

Figure 9:

| 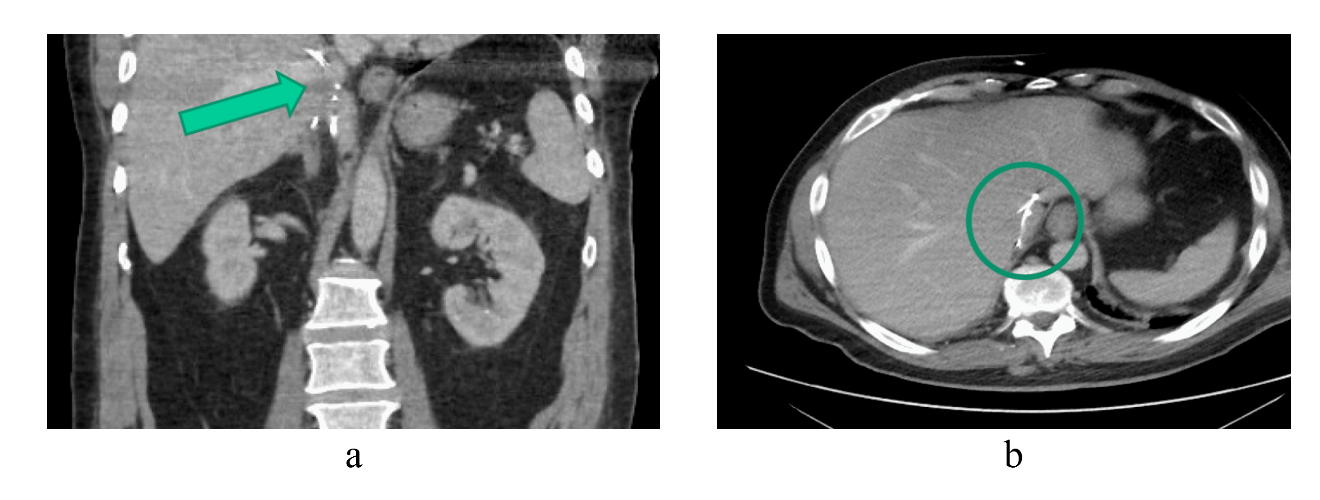 |
| --- |
| Cross-sectional view of IVCF in intrahepatic IVC (a), Coronal view of IVCF in intrahepatic IVC (b). The green mark is the displacement filter.  Image sourced from Gazda ^6^. |

Figure 10:

| 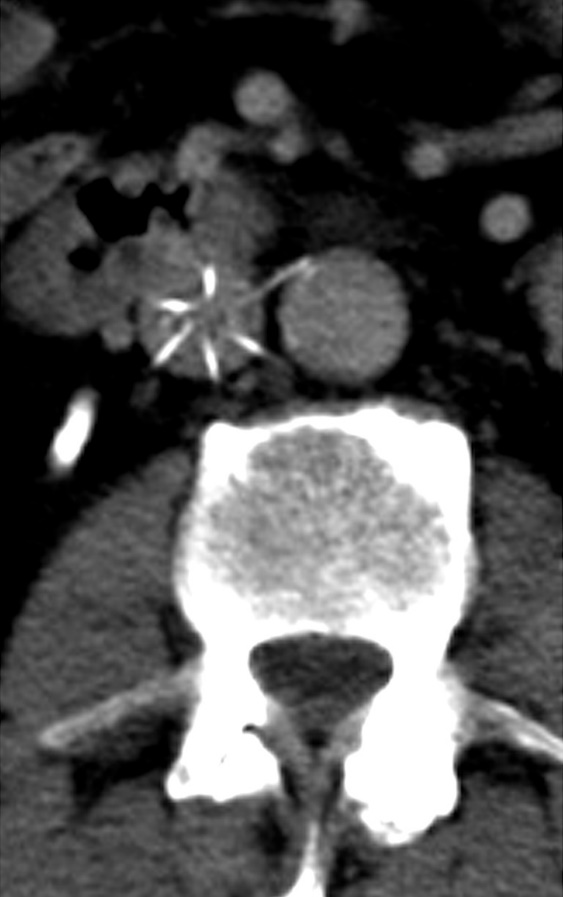 |
| --- |
| Filter perforation. Axial contrast-enhanced CT of the abdomen demonstrates the filter (Recyclable Filter, Bard) perforating the IVC wall with one of its legs in the aortic wall. The patient was asymptomatic. IVC, inferior vena cava.  Image sourced from Grewal ^7^ |

Figure 11:

| 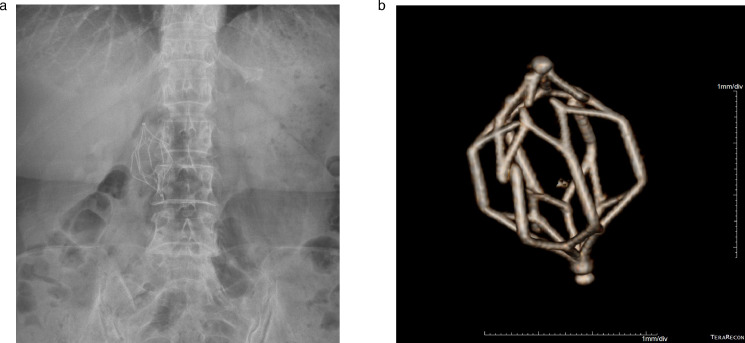 |
| --- |
| Plain abdomen radiograph (a) and the 3D reconstructed image (b) showing the fractured OptEase IVCF in the IVC.  Image sourced from Kim ^8^. |

Figure 12:

| 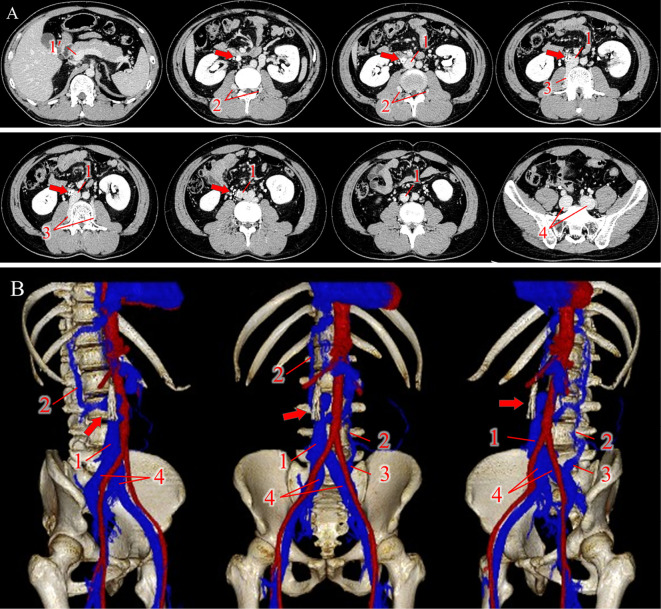 |
| --- |
| A: a CT angiogram on admission showing a dilated and obstructed IVC (1) at the site of the indwelling IVCF (arrow). Blood flow below the site of IVC occlusion returned from the azygos vein to the superior vena cava via the collateral circulation (2, 3). In addition, both sides of the iliac vein (4) were markedly expanded.  B: a 3-dimensional image constructed from the contrast CT images showing the occlusion of the IVC and a detailed view of the collateral circulation. CT: computed tomography, IVC: inferior vena cava, IVCF: inferior vena cava filter. Arrow, IVCF; 1, IVC; 1’, the remaining IVC from the renal vein of the head side; 2, the ascending lumbar vein; 3, the lumbar vein; 4, the iliac vein  Image sourced from Kasai^9^. |

Figure 13:

| 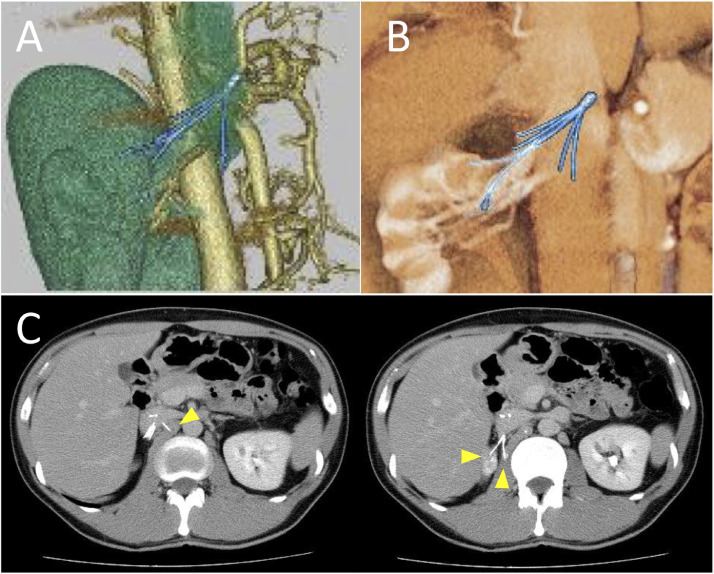 |
| --- |
| Enhanced computed tomography (CT) images one month after implantation of an ALN-type inferior vena cava (IVC) filter. CT images show the first legs of the IVC filter (A, B) incompletely open and greatly tilted and (C) perforating the IVC wall and penetrating the kidney and the iliopsoas muscle (yellow arrowheads).  Image sourced from Shimoo^10^. |

Figure 14:

| 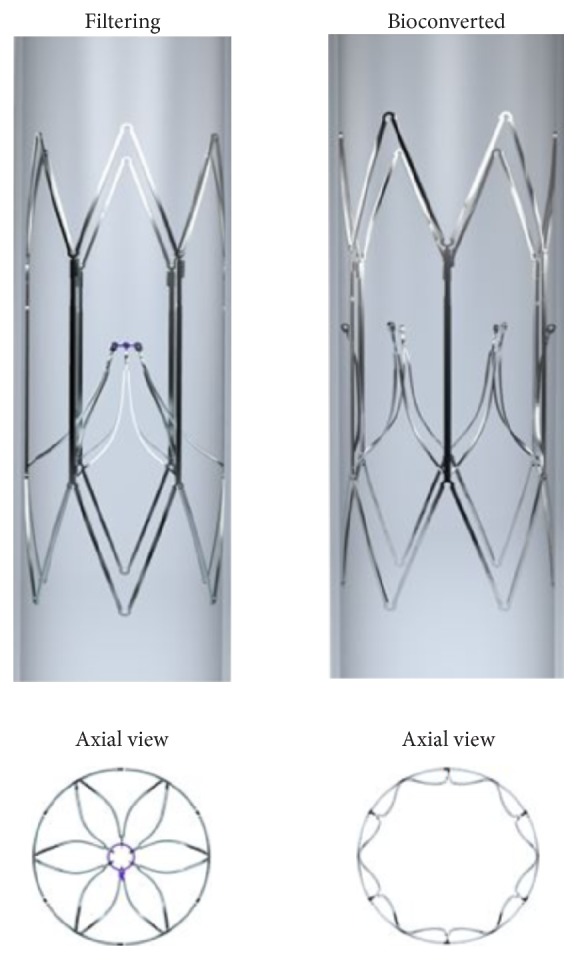 |
| --- |
| Sentry bioconvertible filter’s construction diagram  Image sourced from Gaines ^11^ |

Figure 15:

| 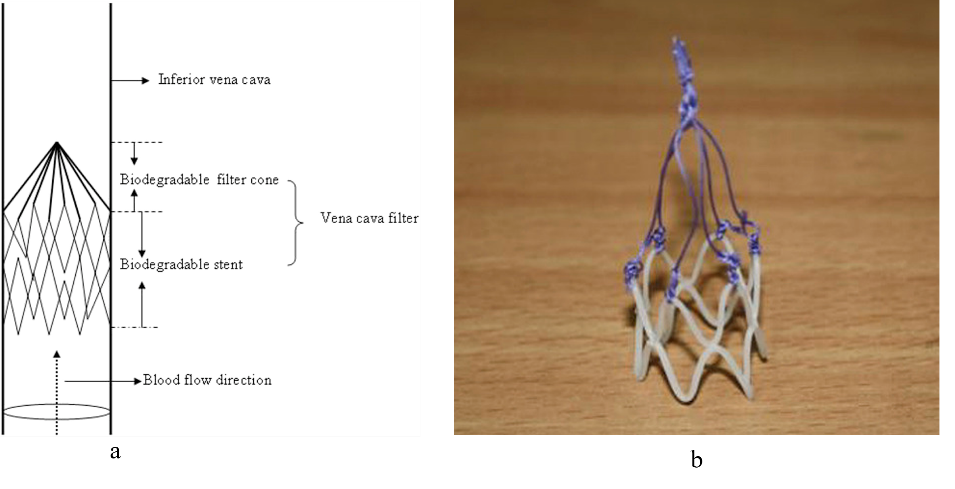 |
| --- |
| a: Schematic drawing of the biodegradable vena cava filter deployed in the vena cava. It consists of two parts, a filter cone and a stent. The filter cone is constructed of six polyglycolic acid polymer strands anchored to an absorbable stent. The absorbable stent, made of polycaprolactone, attaches the filter to the vena cava after deployment.  b: Handmade biodegradable vena cava filter for implantation in canine vena cava.  (a, b) Image sourced from Zhang ^12^ |

1 Li, Z., Ni, R.-f., Zhao, X., Yang, C. & Li, M.-m. Cement embolus trapped in the inferior vena cava filter during percuta neous vertebroplasty. *Korean J Radiol* **14**, 451-454, doi:10.3348/kjr.2013.14.3.451.

2 Prater, S., Awan, M. A., Antuna, K. & Colon, J. Z. Prevention of Pulmonary Cement Embolism by Inferior Vena Cava Filter f ollowing Vertebroplasty-related Cement Intravasation. *J Radiol Case Rep* **15**, 17-27, doi:10.3941/jrcr.v15i4.4139.

3 Han, X., Sheng, Y., Wu, J. & Wang, W. Unretrievable IVC Filter Due to Cement Intravasation. *Cardiovasc Intervent Radiol* **45**, 1048-1050, doi:10.1007/s00270-022-03122-1 (2022).

4 Isaak, A., Takes, M., Kingsmore, D. & Gürke, L. Endovascular Retrieval of Intracaval Cement: A Fishing Net Technique. *Cardiovasc Intervent Radiol* **41**, 1958-1961, doi:10.1007/s00270-018-2061-6.

5 Bardin, F., Bernard, D. & Coudert, M. Duodenal perforation: A rare complication of ALN Optional(®) vena cava filter. *Diagn Interv Imaging* **97**, 943-945, doi:10.1016/j.diii.2014.11.033 (2016).

6 Gazda, A. & Squillante, M. Migration of an Inferior Vena Cava (IVC) Filter Into the Intrahepatic IVC: A Case Report. *Cureus* **14**, e26308, doi:10.7759/cureus.26308 (2022).

7 Grewal, S., Chamarthy, M. R. & Kalva, S. P. Complications of inferior vena cava filters. *Cardiovasc Diagn Ther* **6**, 632-641, doi:10.21037/cdt.2016.09.08 (2016).

8 Kim, J. Inferior vena cava filter fracture and migration to the pulmonary artery. *Radiol Case Rep* **16**, 934-937, doi:10.1016/j.radcr.2021.01.061 (2021).

9 Kasai, H. *et al.* The Development of Marked Collateral Circulation due to Inferior Vena Cava Filter Occlusion in a Patient with Chronic Thromboembolic Pulmonary Hypertension Complicated with Anti-phospholipid Syndrome. *Intern Med* **56**, 931-936, doi:10.2169/internalmedicine.56.7599 (2017).

10 Shimoo, S. & Koide, M. Incomplete opening of an ALN-type inferior vena cava filter due to entanglement of the filter legs resulting in filter migration and inferior vena cava perforation. *Radiol Case Rep* **15**, 1231-1234, doi:10.1016/j.radcr.2020.05.019 (2020).

11 Gaines, P. A. *et al.* Sentry Bioconvertible Inferior Vena Cava Filter: Study of Stages of Incorporation in an Experimental Ovine Model. *Int J Vasc Med* **2018**, 6981505, doi:10.1155/2018/6981505 (2018).

12 Zhang, F., Li, H., Liang, G. & Zhang, H. Development and evaluation of a new biodegradable vena cava filter in a canine model. *Asian J Surg* **40**, 12-16, doi:10.1016/j.asjsur.2015.05.002 (2017).
